# “Glowing Head” Mice: A Genetic Tool Enabling Reliable Preclinical Image-Based Evaluation of Cancers in Immunocompetent Allografts

**DOI:** 10.1371/journal.pone.0109956

**Published:** 2014-11-04

**Authors:** Chi-Ping Day, John Carter, Zoe Weaver Ohler, Carrie Bonomi, Rajaa El Meskini, Philip Martin, Cari Graff-Cherry, Lionel Feigenbaum, Thomas Tüting, Terry Van Dyke, Melinda Hollingshead, Glenn Merlino

**Affiliations:** 1 Laboratory of Cancer Biology and Genetics, National Cancer Institute, Bethesda, Maryland, United States of America; 2 In Vivo Evaluation, Leidos Biomedical Research Inc., Frederick National Laboratory for Cancer Research, Frederick, Maryland, United States of America; 3 Center for Advanced Preclinical Research of The Center for Cancer Research, National Cancer Institute, Frederick, Maryland, United States of America; 4 Center for Advanced Preclinical Research of Leidos Biomedical Research Inc., Frederick National Laboratory for Cancer Research, Frederick, Maryland, United States of America; 5 Laboratory Animal Science Program, Leidos Biomedical Research Inc., Frederick National Laboratory for Cancer Research, Frederick, Maryland, United States of America; 6 Department of Dermatology and Allergy, University Hospital Bonn, Bonn, Germany; 7 Biological Testing Branch, Developmental Therapeutics Program, National Cancer Institute, Frederick, Maryland, United States of America; Virginia Commonwealth University, United States of America

## Abstract

Preclinical therapeutic assessment currently relies on the growth response of established human cell lines xenografted into immunocompromised mice, a strategy that is generally not predictive of clinical outcomes. Immunocompetent genetically engineered mouse (GEM)-derived tumor allograft models offer highly tractable preclinical alternatives and facilitate analysis of clinically promising immunomodulatory agents. Imageable reporters are essential for accurately tracking tumor growth and response, particularly for metastases. Unfortunately, reporters such as luciferase and GFP are foreign antigens in immunocompetent mice, potentially hindering tumor growth and confounding therapeutic responses. Here we assessed the value of reporter-tolerized GEMs as allograft recipients by targeting minimal expression of a luciferase-GFP fusion reporter to the anterior pituitary gland (dubbed the “Glowing Head” or GH mouse). The luciferase-GFP reporter expressed in tumor cells induced adverse immune responses in wildtype mouse, but not in GH mouse, as transplantation hosts. The antigenicity of optical reporters resulted in a decrease in both the growth and metastatic potential of the labeled tumor in wildtype mice as compared to the GH mice. Moreover, reporter expression can also alter the tumor response to chemotherapy or targeted therapy in a context-dependent manner. Thus the GH mice and experimental approaches vetted herein provide concept validation and a strategy for effective, reproducible preclinical evaluation of growth and response kinetics for traceable tumors.

## Introduction

The average drug developed by major pharmaceutical companies has been estimated to cost between 4 and 11 billion dollars [1], costing the average cancer patient approximately $100,000 per year. These staggering costs are driven in part by an inability early in the developmental pipeline to reliably identify drugs that will be efficacious, and the overall approval rate for an oncological compound is currently about 5% [2]. Much of this failure can be attributed to the inadequacy of preclinical models used in therapeutic evaluation. Historically, preclinical animal studies have utilized decades-old established human cell lines, transplanted as xenografts subcutaneously into immunocompromised mice [3]. Unfortunately, these models have had limited efficacy-predictive value for drug development, yet have been deemed critical for improving pharmaceutical productivity and patient care [4].

The proficiency of preclinical cancer studies is linked to the appropriateness of the animal model itself. Paramount is the presence of a fully functional immune system, which is involved in virtually every step of disease development, and critically determines treatment responses [5]. Tumor cells interact reciprocally and dynamically with immune and other microenvironmental cells throughout the course of metastatic progression and also following therapeutic intervention [6]. This interaction is appropriately modeled both in autochthonous genetically engineered mouse (GEM) cancer models and by orthotopic transplantation of GEM-derived allografts (GDAs) into fully immunocompetent host mice [7], but not effectively in current human cancer xenograft models. Finally, therapeutic and biomarker evaluation should ideally rely on preclinical cancer models recapitulating *naturally occurring* metastasis, the most deadly cancer phase.

Tractable preclinical models require the ability to accurately monitor disease progression and therapeutic response, facilitating the adoption of relevant clinical endpoints [8]. Disease monitoring is essential for metastases and otherwise undetectable tumors. Optical imaging of cells expressing light-generating proteins currently dominates monitoring technologies due to their ability to measure real-time events, cost-effectiveness and time-efficiency [9]. However, most traceable marker proteins, including the popular firefly luciferase (ffLuc) and jellyfish enhanced green fluorescent protein (eGFP), are xenobiotic to mammals. Their expression naturally induces various immune responses in immunocompetent animals, resulting in inconsistent activity [10], [11], rejection of grafts [12] and suppression of metastatic activity [13], confounding the validity of preclinical conclusions. Thus, the effective use of xenobiotic reporters is restricted to either short-term studies, or fully immunocompromised animal models, limiting preclinical options [9], [13].

To overcome these problems, we have developed a GEM model that is immune-tolerant to both ffLuc and eGFP to serve as a host for transplantation of labeled syngeneic tumors. Using the rat growth hormone (rGH) promoter, expression of a ffLuc-eGFP fusion protein was targeted to the anterior pituitary, a non-immune privileged site distant from commonly monitored organs in preclinical studies, thereby creating the “Glowing Head” (GH) mouse [14]. We demonstrate that in wildtype mice immune responses induced by xenobiotic reporters substantially affect the progression and therapeutic responses of imageable transplanted tumors. Importantly, the use of pre-tolerized GH mice minimizes or eliminates these aberrations, resulting in more reliable, tractable preclinical models.

## Materials and Methods

### Lentiviral Vectors

The lentiviral vector that expresses the firefly luciferase-enhanced green fluorescent protein fusion protein (FerH-ffLuc-eGFP) was described previously [10]. It was here modified to remove eGFP and insert an internal ribosome binding site (IRES) and histone H2B-tagged eGFP (H2B-eGFP) to generate FerH-ffLuc-IRES-H2B-eGFP, which targets the expression of ffLuc and eGFP to the cytoplasm and nucleus, respectively. Detailed information on the vector sequence will be provided upon request to Dr. Dominic Esposito (e-mail: espositod@mail.nih.gov), Leidos Biomedical Research, Frederick, MD, USA.

### Animals

To reduce bioluminescence absorption and experimental variation, albino 6- to 8-week-old inbred female mice on a C57BL/6 (C57BL/6^c-brd/c-brd^/Cr) or FVB/N background were used as hosts for transplantation studies. F1 mice from the breeding of C57BL/6 with 129 (B6;129) mice were used as isogenic hosts in the study of NRas^Q61K^/p19^ARF^-null melanoma, which was derived from a mixed genetic background [15], [16]. All animals used in this research project were cared for and used humanely according to the following policies: The U.S. Public Health Service Policy on Humane Care and Use of Animals (1996); the Guide for the Care and Use of Laboratory Animals (1996); and the U.S. Government Principles for Utilization and Care of Vertebrate Animals Used in Testing, Research, and Training (1985). All mouse experiments were performed in strict accordance with Animal Study Protocols approved by the Animal Care and Use Committee (ACUC), NCI, at the Frederick National Laboratory for Cancer Research, which is accredited by AAALACi and follows the Public Health Service Policy on the Care and Use of Laboratory Animals. The following protocols were approved by the ACUC for performing this study: ASP# 08–084, 11–044, and 11–058.

The mice in this study were euthanized by CO2 asphyxiation following NCI-approved ACUC guidelines: (1) Transfer the mice to a CO2 chamber right before euthanasia. (2) Turn on the CO2 at 2 liters per minute for a standard sized of chamber. (3) Within approximately two to three minutes, adult mice should be immobile and unresponsive; when this is evident, increase the flow rate to high or approximately 10 liters/min. (4) When breathing ceases for all animals seen through the cage, set the timer for 2 minutes. At the end of two minutes, the mice may be removed from the CO2-filled cage. Ensure death by making sure there are no movements of any kind for an additional 60 seconds outside the CO2-filled cage, using the timer.

### Generation of the “Glowing Head” mouse

The rGH-hGH construct [17] (a gift of Dr. Rhonda Kineman, University of Illinois-Chicago, Chicago, IL) was modified by insertion of an ffLuc-eGFP fusion gene to generate the anterior pituitary gland-targeting vector, which was used to generate transgenic mice in both the C57BL/6 and FVB/N genetic backgrounds by blastocyst microinjection. Small colonies of homozygous transgenic mice were maintained for breeding purposes, and their heterozygous progeny used for all preclinical studies. All the transgenic and breeding work was performed through the Laboratory Animal Science Program, Frederick National Laboratory.

### Murine tumors, cancer cell lines, and their labeling

The Lewis Lung Carcinoma (LLC) tissue was maintained only *in vivo* since its derivation from the original lung tumor of C57BL/6 mice [8]. The spontaneously metastasizing serial Hgf-tg/CDK4^R24C^ melanoma skin transplant was generated from a primary melanoma induced in Hgf-tg/CDK4^R24C^ C57BL/6 mice by epicutaneous application of the carcinogen DMBA [18]. HGF-tg/CDKN2A^-/-^ melanoma was derived from tumors induced in HGF-tg/CDKN2A^-/-^ FVB mice by UV irradiation [19]. These tumors were maintained only in syngeneic mice. For transplantation, the harvested tumor tissues were divided into 3 mm ×3 mm pieces and each one was inserted into a 5-mm cut on skin of a mouse. Mvt-1 murine breast cancer cells were derived from mammary tumors of the MMTV-c-Myc/MMTV-Vegf bi-transgenic mouse on an FVB/N inbred background [20]. They were established as a cell line and maintained through in vitro culture. For transplantation, 1.0×10^6^ cells were prepared from culture and injected subcutaneously into each mouse. Mutant NRas^Q61K^/p19^ARF^-null melanoma cells were generated as described [15], [16]. In the first passage, 1.0×106 cells from in vitro culture were inoculated into C57BL/6x129 F1 mice to form tumors. In the following passages, the fragments divided from harvested tumor were used for transplantation, as described above.

To label the *in vivo* maintained tumors, cell suspensions prepared from *in vivo*-expanded tumors were infected *ex vivo* with lentivirus by *ex vivo* spinoculation [10], [21]. LLC tissue was infected with lentivirus encoding ffLuc-eGFP or ffLuc-IRES-H2B-eGFP and then subjected to *in vivo* cycling to obtain uniformly-labeled tumors, as described previously [8]. Cell lines were labeled with ffLuc-eGFP lentivirus *in vitro*, and the eGFP^+^ populations were isolated using the fluorescence-activated cell sorter (FACS).

### Preclinical studies and pathological analysis

For preclinical studies, a cryogenically preserved labeled tumor was revived and expanded by subcutaneous transplantation into mice. These tumors were resected upon reaching 500 mm^3^ and expanded through passage into the requisite number of mice for the actual studies described in the text. Tumor size was measured manually and calculated by V (mm^3^)  = 0.5×L×W^2^, where L is length and W is width in mm. For the preclinical modeling of primary tumors, mice were randomized into groups according to study design when their tumors reached 125 mm^3^. The control group received vehicle solution, and the experimental group received treatments of chemotherapeutic agents. The dose and schedule in each experiment have been specified in the Results. When tumors grew to 2000 mm^3^, mice had reached their endpoints and were euthanized for further study.

For preclinical models of spontaneous metastasis, primary tumors were surgically removed upon reaching 500 mm^3^, and the mice were randomized into groups according to the study design. Metastasis and recurrence were monitored periodically by imaging using the Xenogen IVIS system [8] to measure BL flux (photon/sec/radial degree). The control group received vehicle solution, and the experimental group received treatments of chemotherapeutic agents. The dose and schedule in each experiment have been specified in the Results. When mice showed signs of morbidity, defined by the animal study protocol (e.g. short of breathiness, difficulty in moving), they reached their endpoint and were euthanized for further study.

The drugs used in this study were obtained from the Drug Synthesis & Chemistry Branch, DTP, NCI (Bethesda, MD). Paclitaxel was dissolved at 10x the desired concentration in 100% ethanol, diluted with an equal volume of Cremaphor EL and then diluted to the 1x concentration with saline before intravenous injection into mice. Gemcitabine was dissolved in water and injected intraperitoneally into mice. Crizotinib was resuspended in 0.5% methylcellulose in 0.9% saline, and given once daily by oral gavage (PO) over a 3-week period at 10 ml/Kg. Mice carrying subcutaneous tumors were randomized into 3 groups based on tumor measurement (200–500 mm^3^), and treated with vehicle alone, crizotinib at 50 mg/kg, or Crizotinib at 100 mg/kg.

Harvested tissues were fixed in 10% formaldehyde and paraffin-embedded. Adjacent serial sections were stained with hematoxylin and eosin (H&E) for histological analysis, or used for GFP immunohistochemistry (ab6556, Abcam, Cambridge, MA, USA). Histopathology was performed by Dr. Miriam Anver (Pathology and Histotechnology Laboratory, Leidos Biomedical Research, Frederick, MD). For quantitative analysis, slides were scanned using the ScanScope XT system and images were analyzed by Spectrum Plus pathology analysis software (Aperio Technologies, Vista, CA).

### Hormone and immunological marker analysis

Sera were prepared from the collected whole blood following conventional protocols and stored at −80°C. To analyze anti-GFP antibody in serum, ELISA plates (Nunc MaxiSorp, cat# 439454, Thermo Scientific, Waltham, MA, USA) were coated with 31.25 ng of recombinant GFP (MB-0752, Vector Laboratory, Burlingame, CA, USA) in each well overnight at 4°C. The next day, sera and control monoclonal anti-GFP antibody (11814460001, Roche Applied Science, Indianapolis, IN, USA) were subjected to serial dilution with blocking solution (3% milk in phosphate-buffered saline [PBS]) to reach the range 1∶25–1∶2000 for the former and 6.25–200 ng/ml for the latter. 50 µl of diluted sera or control antibody were added to the coated wells, followed by incubation for an hour at room temperature. After washing with PBS containing 0.05% Tween 20 (PBST). Horse reddish peroxide (HRP)-conjugated goat anti-mouse antibody (115-035-062, Jackson ImmunoResearch Laboratories) at 1∶1000 dilution in blocking solution was then added into each well, followed by the addition of peroxidase substrate (TMB 2-Component Microwell Peroxidase Substrate Kit, 50-76-00, KPL, Gaithersburg, MD, USA) for color development according to the manufacturer's instruction. The A450 absorption of the plates was measured using a microplate reader (VMax Kinetic ELISA Absorbance Microplate Reader, 97059-546, VWR Corp., Radnor, PA, USA). Mouse growth hormone levels in sera were analyzed using the Growth Hormone (GH) ELISA kit (M0934, Biotang Inc., CA, USA) according to the manufacturer's instruction as following. Sera were diluted 2-fold with RPMI1640 medium, and standard solutions were prepared for the concentration range 0.3125–100 ng/ml. The standards and samples were added into the provided ELISA plate, which was incubated at 37°C for 40 min and washed with washing buffer. Each well was then added with 50 µl of water and 50 µl of biotinylated anti-GH antibody, and incubated at 37°C for 20 min. After washing, 100 µl of streptavidin-conjugated HRP was added into each well and incubated at 37°C for 10 min. After another washing, 100 µl of HRP substrate solution was added to each well, incubated at 37°C for 15 min, followed by adding 100 µl of stop solution. The A450 absorption of the plates was measured using the VMax microplate reader.

To analyze cell surface markers, single-cell suspensions were prepared from harvested mouse spleens and incubated with 5 µl/ml of Fcγ Receptor antibody (14-0161-85, eBiosciences, San Diego, CA, USA) for blocking for 20 min. Following a wash with staining solution (PBS containing 1% bovine serum albumin [BSA]), they were incubated with 0.3 µl/ml of rat anti-mouse CD4 (550728, BD-Pharmingen, San Jose, CA, USA) or CD8α (550281, BD-Pharmingen antibody, or isotype control antibody (559073, BD-Pharmingen) at 4°C for 1 hr, followed by washing with staining solution for three times. The cells were then incubated with 4 µl/ml of Alexa 488-conjugated goat anti-rat secondary antibody (A11006, Invitrogen, Grand Island, NY, USA) at 4°C for 20 min. After washing with staining solution for three times, the cells were subjected to FACS analysis (FACSCalibur, BD Biosciences, San Jose, CA, USA) or Cell Analyzer equipped with a filter optics module for FITC detection to quantitate the expression of cell markers (Cellometer Vision, Nexcelom Bioscience, Lawrence, MA, USA). The data generated from FACS and Cellometer Vision were analyzed and quantitated with software FlowJo (TreeStar, Inc. Ashland, OR, USA) and FCS Express (De Novo Software, Los Angeles, CA, USA), respectively.

### Statistical analysis

Differences in quantity distribution (e.g. tumor size, bioluminescence intensity, CD8/CD4 ratio) between study groups were analyzed using the parametric unpaired t test. For preclinical studies, the end point was overall survival, defined as the time until mouse morbidity according to the animal study protocol. Mice alive at the end of the study were censored at that date. The Kaplan-Meier method and Mantel-Cox logrank-test were performed to compare survival rates of the mouse groups. Statistical significance was established at the *P*-value <0.05. The median survival time was calculated as the smallest survival time for which the survivor function reached 50%. The computations were done with GraphPad Prism 6 (La Jolla, CA).

## Results

### Reporter activity of ffLuc-eGFP-labeled murine tumors is inconsistent in immunocompetent syngeneic mice

**Figure 1:**
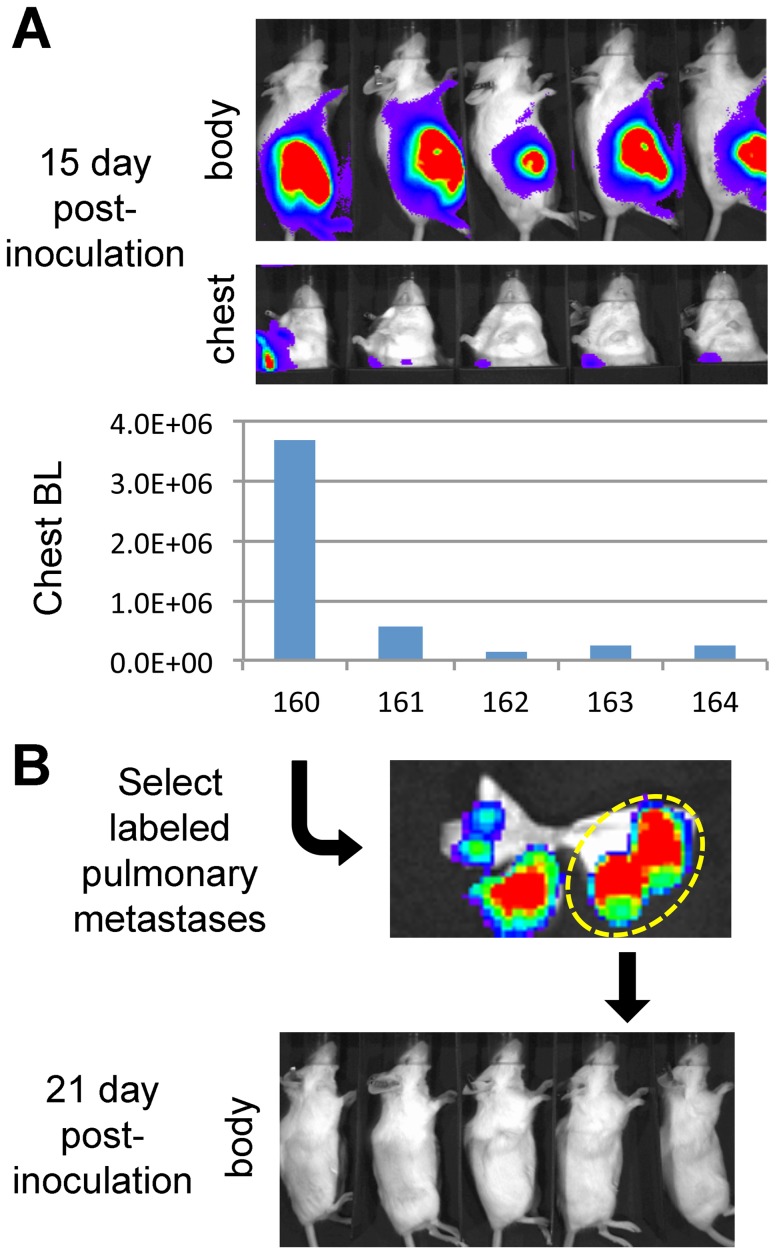
Inconsistency of ffLuc-eGFP reporter activity in labeled tumors during passages in syngeneic immunocompetent mice. **A**, Murine Lewis Lung Carcinoma (LLC) cells were infected with ffLuc-eGFP-expressing lentivirus *ex vivo*, and subcutaneously transplanted into five syngeneic albino C57BL/6 (c-Brd) mice (#160–164). Reporter activity was monitored by BL imaging of subcutaneous tumor growth (body) and pulmonary metastasis (chest). At day 15 after inoculation, a metastatic BL signal was found in one of the mice (#160 in the lower panel). **B**, The lung was harvested from #160, and a single glowing metastatic nodule selected using *ex vivo* imaging (upper panel) was transplanted into five c-Brd mice in the second passage. Imaging results showed that the reporter activity could not be consistently maintained in the resulting palpable tumors (lower panel).

The subcutaneously transplanted Lewis Lung Carcinoma (LLC) is a well-characterized metastatic model that has recently been exploited in several high profile preclinical studies [22]–[24]. We recently retrieved archived LLC tissue never adapted to cell culture, and showed that following transplantation and resection metastasis occurred with very short latency in >90% of syngeneic WT C57BL/6 host mice [8]. Here we labeled LLC with an ffLuc-eGFP-encoded lentivirus *ex vivo* [10]. Since viral transduction results in heterogeneous cell population [25], we subject this labeled tumor to *in vivo* cycling to render them uniformly labeled [8], [26]. Briefly, mice bearing transplanted tumors are monitored for metastasis, and metastatic nodules will be harvested for subcutaneous transplantation to initiate next cycle. Since each nodule was derived from a single cell, the tumor derived from it is presumably clonal. Therefore, homogeneity will be enhanced through each cycle. As shown in Fig. 1A, following subcutaneously transplantation and resection of the labeled LLC in five mice, arising metastases were readily detected by *in vivo* bioluminescence (BL) imaging. In this passage, although tumors grew in all hosts, metastases were detected in only one (#160 in Fig. 1A, lower panel). We harvested lungs from that mouse and examined it with *ex vivo* imaging (Fig. 1B, upper panel). The unevenly distributed BL intensity reflected the heterogeneity of transduced cells in primary tumor (Fig. 1B, upper panel). We collected from host mice three individual well-labeled lung metastases, presumed to be clonal, dividing them into five fragments for transplantation into five C57BL/6 mice. Labeled pulmonary nodules from that mouse were collected and transplanted into another five C57BL/6 mice; however, these tumors then grew very slowly and/or exhibited no detectable reporter activity (Fig. 1B). These results demonstrate that reporter activity in labeled cells could not be consistently maintained over passages in syngeneic immunocompetent mice, even after clonal selection.

To determine if reporter consistency was dependent on tumor type, we extended our analysis to mouse melanoma. An NRas^Q61K^-transformed, p19^ARF^-deficient melanocytic cell line [15], [16] was labeled using the ffLuc-eGFP lentivirus and transplanted subcutaneously into syngeneic immunocompetent mice. Following resection one high-BL pulmonary nodule was selected for subcutaneous transplantation into two mice (Fig. S1A, left panels). Both tumors exhibited a significant reduction in normalized BL activity during subcutaneous growth (Fig. S1A, right panels). We corroborated these results in two other models. Melanoma cells harvested directly from an HGF-transgenic/CDKN2A-knockout FVB/N mouse were transduced with the ffLuc-eGFP gene *ex vivo*, and transplanted subcutaneously into syngeneic FVB/N mice. While all tumors grew, BL intensity was either reduced or increased more slowly (Fig. S1B), and BL intensity/size ratio, serving as the labeling retention indicator, was reduced in three of five tumors. In another model, ffLuc-eGFP-expressing mouse Mvt-1 breast cancer cells transplanted orthotopically into mammary fat pads of syngeneic FVB/N mice also demonstrated poor retention of BL signaling (see below). We conclude that ffLuc-eGFP expression in allografts in immunocompetent wildtype (WT) mice is inconsistently maintained between mice and/or passages, irrespective of tumor type, genetic background or transplantation site.

### Generation of a GEM model immunologically tolerant to GFP and luciferase reporters

**Figure 2:**
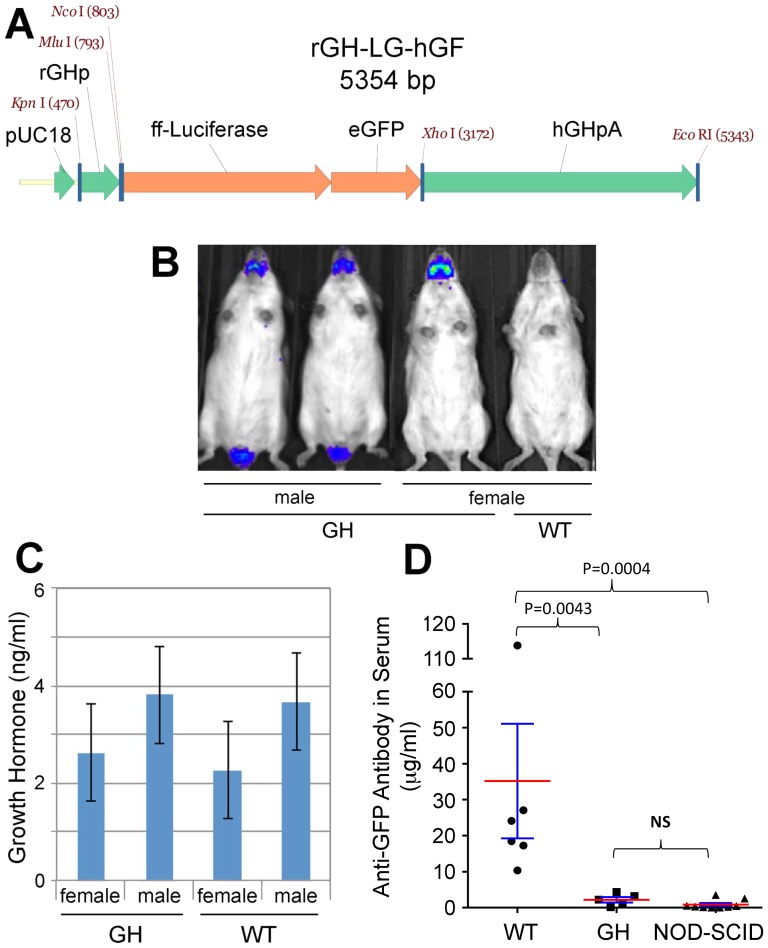
Generation of the rGH-ffLuc-eGFP (“Glowing Head”) genetically engineered mouse. **A**, Structure of the expression vector for generation of Glowing Head (GH) transgenic mice. Expression of a firefly luciferase-eGFP fusion gene (ffLuc-eGFP) was targeted to the mouse anterior pituitary gland by using the rat growth hormone promoter (rGH) and human growth hormone gene sequences, which include a polyadenylylation site (hGHpA)^20^. **B**, Optical expression pattern of transgene in GH mice as visualized by BL imaging. Reporter activity was detected in the anterior pituitary gland of both genders and the testes of male mice. **C**, Serum levels of growth hormone from age-matched GH mice and wildtype (WT) c-Brd mice was assessed by ELISA (mean ± SE). Blood was withdrawn at the same time of day. No significant differences in circulating growth hormone levels between the GH and WT mice were found. **D**, ffLuc-eGFP-labeled LLC tumors were subcutaneously transplanted into WT, GH, and NOD-SCID mice. Blood was withdrawn to prepare sera when tumors reached 500 mm^3^, and the serum levels of anti-GFP antibody were analyzed by ELISA. The levels of anti-GFP antibody in WT mice are significantly higher than those in GH and NOD-SCID mice (p<0.005), but no difference was found between those in GH and NOD-SCID mice (p = 0.19). The sera from healthy mice without tumor transplantation served as controls to define zero point.

Our results suggested that immunogenicity of xenobiotic reporter gene products is largely responsible for their inconsistency in the context of a fully functional immune system. To circumvent this issue, we generated C57BL/6- and FVB/N-based GEM models recognizing ffLuc and eGFP proteins as self. For its high specificity, rGH gene sequences [17] (Fig. 2A) were employed to target expression of an ffLuc-eGFP fusion gene to the anterior pituitary gland of the mouse, thereby avoiding interfering signaling from the most common metastatic sites.

The anterior pituitary gland is not an immune-privileged site and is thus part of systemic circulation [27]. The transgene-encoded ffLuc and eGFP proteins expressed in the anterior pituitary gland during embryonic development therefore participate in the selection of T and B cells and are recognized as self-antigens, resulting in their tolerization. To reduce light adsorption by pigment the ffLuc-eGFP transgene was bred into the albino C57BL/6F (c-Brd) background. Founder lines were chosen from each strain that demonstrated Mendelian transgene inheritance and normal fecundity. In our previous study, we identified the detection limit of BL signal from in vivo mouse imaging was 1.5×10^5^ photon/sec/rad [8]. To avoid possible confounding effects associated with high transgene expression, those founder lines exhibiting low but consistent BL signal above background reading (about 2–6×10^5^ photon/sec/rad) were selected (Fig. S2A). Consistent with targeting reported for the rGH promoter [17], BL signal was evident in the head and testes of transgenic lines (Fig. 2B); modest signal could also be detected in the thyroid glands, but only by using *ex vivo* imaging (not shown). The BL levels in the body of GH mice are close to those in WT mice, indicating the high specificity of the transgene (Fig. S2B). Based on the site of reporter activity and rGH promoter used for targeting these GEMs were dubbed “Glowing Head” (GH) mice.

The possible impact of transgene expression on pituitary function was evaluated by comparing circulating growth hormone levels in GH and WT C57BL/6 mice. We found that serum growth hormone levels were not significantly different between transgenic and WT (Fig. 2C), irrespective of gender, indicating that expression of the ffLuc-eGFP transgene does not overtly affect anterior pituitary function in GH mice.

To assess the immunological consequences of reporter expression, cells from ffLuc-eGFP-labeled LLC tumors were transplanted subcutaneously into GH and WT C57BL/6 mice, as well as MHC-unmatched, immunocompromised non-obese diabetic/severe combined immunodeficiency (NOD/SCID) mice (BALB/c background). When tumors reached 500 mm^3^ blood was withdrawn and sera tested for the presence of anti-GFP antibody. While tumor-bearing WT mice possessed significant levels of circulating anti-GFP antibody (Fig. 2D and Fig. S2C), no significant difference was found between tumor-bearing GH and NOD-SCID mice, which is known incompetent to produce antibody (Fig. S2C). These data show that while immunogenic in WT mice, ffLuc-eGFP is tolerated and recognized as self in GH mice.

### Growth and metastasis of tumor cells expressing imageable xenobiotic reporters are altered in WT and NOD/SCID mice compared to GH mice

**Figure 3:**
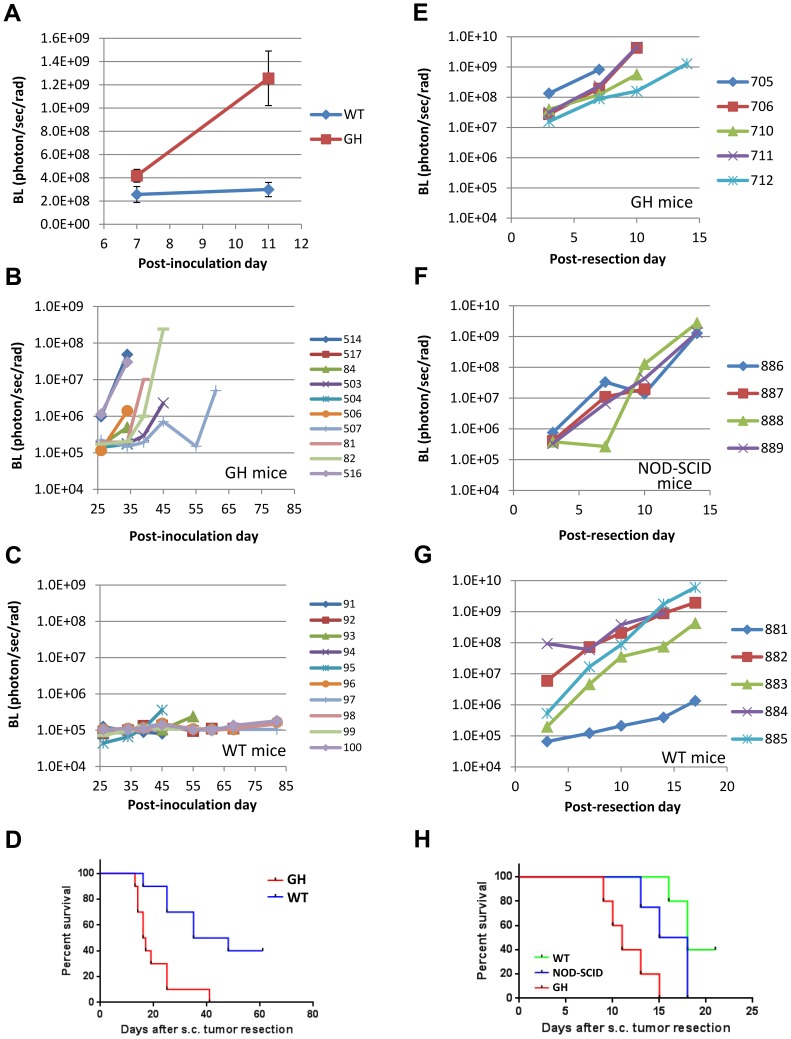
Reporter activity and metastasis of ffLuc-eGFP-labeled cancer cells are consistent in GH mice but suppressed in immunocompetent wildtype mice. **A–D**, Functional comparison of GH and WT mice as transplantation hosts using a breast cancer model. The GFP^+^ population from ffLuc-eGFP-transduced Mvt1 mouse breast cancer cells was isolated and expanded in culture. 1×10^5^ cells were injected into the mammary fat pads (m.f.p.) of WT and GH syngeneic FVB/N mice, followed by BL imaging to monitor tumor growth. Though tumors grew in the fat pads of both groups, the BL intensity (mean ± SE) of those in WT mice was highly suppressed relative to GH mice (**A**). *, *P* = 0.083; **, *P*<0.001. (**B–C**) Upon reaching 500 mm^3^ m.f.p. tumors were resected, and BL imaging was used to monitor metastatic progression, which is visualized by body BL signal in each mouse. Metastatic disease progressed consistently in GH mice (**B**), while being suppressed in WT mice (**C**); the sign and number at side refer to individual mice in each figure. Kaplan-Meier survival analysis showed that GH mice exhibited significantly shorter survival times than WT mice (*P* = 0.0025). Median survival times in GH and WT groups were 16.5 and 41.5 days, respectively (**D**). **E–H**, Behavioral inconsistency of labeled tumors in WT and immunocompromised mice as compared to GH mice. ffLuc-eGFP-labeled LLC tumors were transplanted subcutaneously into syngeneic GH mice, strain-unmatched immunocompromised NOD/SCID (BALB/c) mice, and syngeneic c-Brd (WT) mice. Upon reaching 500 mm^3^ subcutaneous tumors were resected, and mice were subjected to periodic BL imaging to monitor metastasis. The growth curves representing metastatic growth in GH (**E**), NOD/SCID (**F**), and c-Brd WT mice (**G**) are shown; the sign and number at side refer to individual mice in each figure. Compared to those in GH mice, the metastatic growth in the other two groups exhibited heterogeneous and delayed patterns. In accordance with their more efficient metastatic progression, Kaplan-Meyer survival analysis showed that GH mice exhibited significantly shorter survival time than the other two strains of mice (*P* = 0.0037). Median survival times in WT, NOD/SCID, and GH groups were 18 days, 16.5 days and 11 days, respectively (**H**).

To test the function of GH mice, we implanted ffLuc-EGFP-labeled tumors subcutaneously into syngeneic WT and GH mice. Although tumor size increased similarly in both types of host mice, BL increases in tumors were significantly delayed in WT mice as compared to GH mice (Fig. S3A). This result suggested that using GH mice as allograft recipients could help correct the inconsistencies observed in BL signals from labeled tumors transplanted into immunocompetent mice. To validate this point, we tested GH mouse in a larger scale study involving both primary tumor and metastasis. Metastatic Mvt-1 breast cancer cells [20], [28] were transduced with ffLuc-eGFP lentivirus and transplanted orthotopically into mammary fat pads of GH or WT syngeneic FVB/N recipient mice. Labeled Mvt-1 cells exhibited a significant enhancement in BL signaling over time when transplanted into GH mice vs. WT, which failed to retain signaling (Fig. 3A and higher panels of Fig. S4A). Since imageable reporters are essential for monitoring metastasis, responsible for the vast majority of cancer patient deaths, primary Mvt-1 tumors were resected and host mice followed over time. BL imaging showed that metastases were present after a few days and grew efficiently in GH mice (Fig. 3B and lower right panels of Fig. S4A). In contrast, metastases were first detected in a small percentage of WT mice at day 20, while most mice remained BL-free for over 2 months (Fig. 3C and lower left panel of Fig. S4A). Notably, at the experimental endpoint *ex vivo* imaging revealed that metastases were found at multiple sites in GH mice, but only in the lungs of WT mice (Fig. S4B). The survival of WT mice was also significantly prolonged compared to GH mice (Fig. 3D; p = 0.0025). These results indicate that immunity against xenobiotic reporters can suppress the metastatic potential of transplanted labeled cancer cells, and highlight the advantages provided by the GH mouse for monitoring cancer progression and cell tracking.

We corroborated and expanded our assessment of the GH mouse using ffLuc-eGFP-expressing LLC cells. Well-labeled LLC cells were transplanted subcutaneously into GH, WT and also NOD/SCID mice, which have residual innate immune activity, and arising tumors resected at the same size. In the first imaging after resection (day 3 in Fig. 3E to 3G and Fig. S5), metastases arose with higher BL levels in GH mice relative to those in WT and NOD/SCID mice. Subsequent monitoring revealed that metastases progressed efficiently and caused the death of all GH mice from day 9 to 15. As compared to GH mice, the overall disease progression was delayed in NOD/SCID and even more in WT mice. Accordingly, all the NOD/SCID mice died from day 13 to 18, while two of five WT mice were still alive at day 18 (Fig. S5). Importantly, the median survival time of GH mice was significantly shorter than that of either WT or NOD/SCID mice (Fig. 3H; *P* = 0.0037). These results demonstrate that immune responses against xenobiotic reporters can restrict the growth and metastatic potential of labeled tumors in immunocompetent and even partly immunocompromised mice, a problem that could be overcome through the use of GH host mice.

### Immunogenicity associated with imageable reporter expression influences the therapeutic outcome of preclinical mouse studies

**Figure 4:**
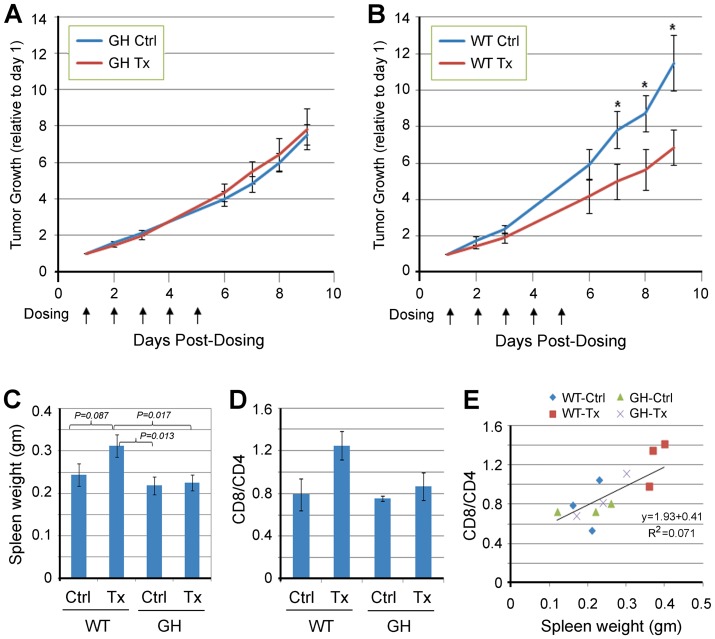
Immunogenicity of ffLuc-eGFP alters the response of tumors to chemotherapeutic agents in wildtype mice compared to GH mice. Labeled LLC tumors were inoculated subcutaneously into WT and GH c-Brd mice. When the average tumor size reached 125 mm^3^, each strain of mice was randomized into two groups to receive either control vehicle (Cremophor EL + saline) or paclitaxel. Tumor size was measured periodically. **A and B**, Tumor growth (fold-increase relative to day 1) in WT and GH c-Brd mice (mean ± SE). Paclitaxel treatment was inefficacious in GH mice (**A**), but delayed tumor growth in WT mice (*, *P*<0.05 in a two-tailed T-test) (**B**). Ctrl, control vehicle; Tx, paclitaxel treatment. **C**, Spleen size in each group (mean ± SE). Spleens in paclitaxel-treated WT c-Brd mice were marginally bigger than those in vehicle-treated c-Brd mice but significantly bigger than those in both groups of GH mice. No significant difference was found between the two GH mouse groups. **D and E**, Enlarged spleens in paclitaxel-treated WT mice correspond to higher CD8/CD4 ratios. Splenocytes were prepared from spleens harvested from mice from each treatment group. These were stained with anti-mouse CD4 or CD8 antibodies, and analyzed by flow cytometry and Cellometer to obtain the ratio of the CD8+ to CD4+ subpopulation (CD8/CD4) in WT and GH c-Brd host mice (mean ± SE) (**D**). **E**, Regressional analysis demonstrated a significant correlation between CD8/CD4 ratio and spleen size (*P*<0.01).

The advantages illustrated above suggest that GH mice would constitute a superior preclinical model for drug assessment. We have shown that chemotherapeutic paclitaxel has no significant effect on growth of subcutaneous LLC tumors in syngeneic C57BL/6 hosts, irrespective of doses ranging between 6.7–22 mg/kg, QDx5 (Fig. S6). In this study syngeneic GH and WT mice carrying subcutaneous ffLuc-eGFP-labeled LLC tumors were randomized to receive vehicle or paclitaxel at 7.5 mg/kg, QDx5, considered to be a dose mimicking human treatment [8], [29]. As with unlabeled LLC growing in WT mice, paclitaxel had no effect on tumors growing in GH mice (Fig. 4A); in contrast, growth of the ffLuc-eGFP-labeled tumor was significantly delayed in treated WT mice (Fig. 4B). Interestingly, the spleens of paclitaxel-treated WT mice were significantly larger relative to the other three groups (Fig. 4C), and exhibited enlarged, disrupted lymphatic follicles (Fig. S7). Accordingly, the CD8/CD4 ratio of splenocytes increased in paclitaxel-treated WT mice (Fig. 4D), correlating with spleen size in all groups (Fig. 4E). There was no difference in the growth or response to paclitaxel of unlabeled LLC cells growing in WT vs. GH mice (not shown). These data suggest that paclitaxel treatment could produce a false-positive preclinical outcome by inducing a cytotoxic T cell response against a xenobiotic tumor antigen, but only in WT mice that had not been pre-tolerized to that antigen. Taken more broadly, our results show that tumor antigens can significantly influence preclinical tumor response to chemotherapy.

**Figure 5:**
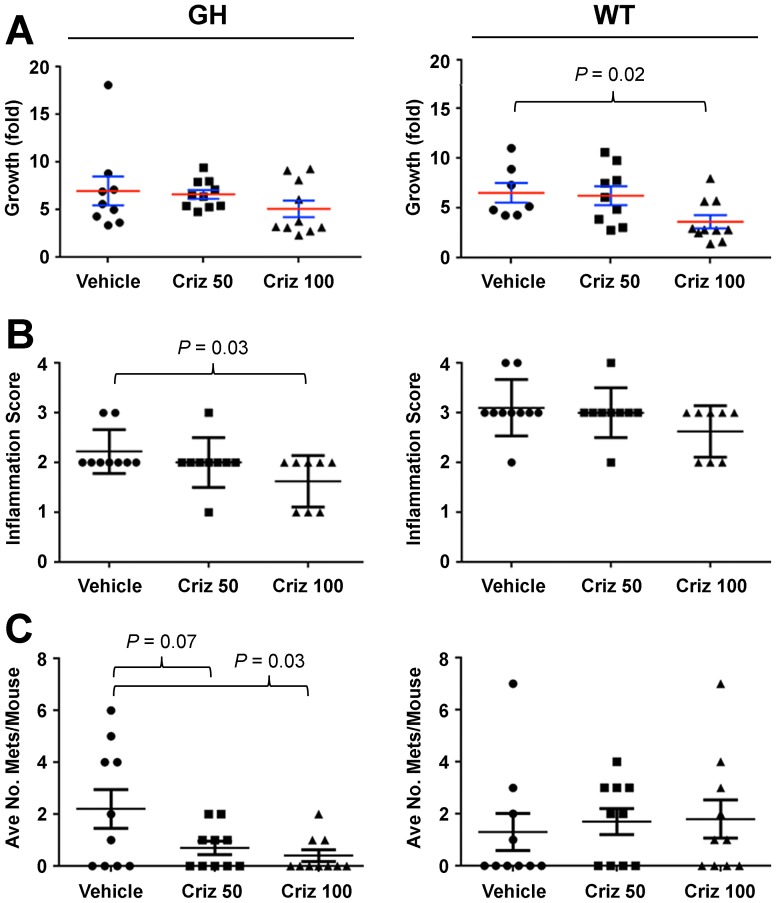
Immunogenicity of ffLuc-eGFP alters the response of tumors, including metastases, to targeted therapy in wildtype mice compared to GH mice. Labeled melanoma tissues from the HGF/CDK4^R24C^-transgenic mouse [18] were subcutaneously inoculated into WT and GH c-Brd mice. When the average tumor size reached 125 mm^3^, mice from each strain were randomized into two groups to receive either control vehicle (saline) or the MET inhibitor crizotinib (Criz). Tumor size was measured periodically. **A**, Fold-tumor growth in WT and GH c-Brd mice (mean ± SE). 100 mg/kg Criz treatment delayed tumor growth in WT mice (right panel, *P*<0.02 in two-tailed T-test), but no efficacy was found in GH mice (left panel). **B**, When primary tumors reached 2000 mm^3^, the mice were euthanized to harvest tumors and lungs. Tumors were subjected to pathological analysis of inflammatory infiltrates according to the scoring system: minimal  = 1, mild  = 2, moderate  = 3, severe  = 4. Criz at 100 mg/kg significantly reduced inflammatory infiltration of the primary tumors in GH mice (left panel), but not in WT mice (right panel). **C**, The fixed lungs were subjected to the quantitation of metastatic foci. Criz reduced the number of pulmonary metastases in GH mice in a dose-dependent manner (left panel), but had no effect in WT mice (right panel).

To assess the effects of antigenic reporters on response to molecularly-targeted therapeutic agents, we employed the melanoma GDA model HCmel12 (derived from an HGF/CDK4^R24C^-transgenic mouse [18]), labeled *ex vivo* with ffLuc-eGFP, and transplanted subcutaneously into syngeneic GH or WT c-Brd mice. Upon reaching 125 mm^3^, mice were randomized to receive either vehicle or crizotinib, a drug targeting the HGF receptor (MET). Crizotinib effected insignificant or modest changes on tumor growth in GH and WT recipients, respectively (Fig. 5A). Pathological analysis revealed that in GH, but not WT, host mice crizotinib significantly reduced inflammation and tumor invasiveness at the primary site (Fig. 5B and Fig. S8). Moreover, crizotinib significantly reduced the number of pulmonary metastases in a dose-dependent manner only in GH mice (Fig. 5C). In this case, our data indicate that immunity against xenobiotic reporters can produce a false-negative response in WT mice, which can be avoided by using GH mice as hosts.

### GH mice enable the ability to reliably track metastatic disease progression and therapeutic response in fully immunocompetent preclinical models

**Figure 6:**
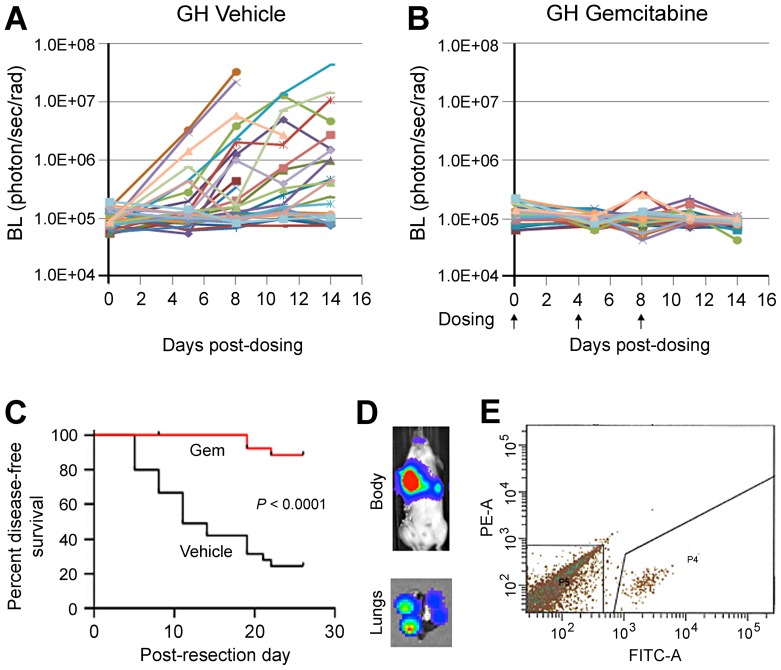
The GH mouse allows for consistent tracking of the progression of labeled metastases, their therapeutic responses and isolation in a preclinical adjuvant study. ffLuc-eGFP-labeled LLC tumors were transplanted subcutaneously into GH mice. Upon reaching 500 mm^3^, the primary tumors were resected. **A and B**, GH mice were randomized into control and treated groups to receive vehicle and gemcitabine at 25 mg/kg, respectively. Metastatic progression in mice was periodically monitored by BL imaging. Metastatic growth was efficient in untreated GH mice (**A**) but suppressed by gemcitabine (**B**). **C**, Kaplan-Meyer analysis showed that survival was significantly shorter in control compared to treated GH mice. **D and E**, GFP+ cancer cells can be readily isolated from whole lungs of GH mice. The lungs were harvested from control GH mice, made into a single-cell suspension, and subjected to sorting by FACS to isolate GFP^+^ cells. The representative result from mouse #806 is shown. The *in vivo* image of mice and *ex vivo* image of lung (**D**) showed BL signal from pulmonary metastases and individual lung nodules, respectively. The GFP^+^ cancer cells were successfully isolated from whole lung by FACS sorting (P4 subpopulation in **E**).

Previously, we demonstrated the feasibility of tracking cancer recurrence and progression with BL imaging in metastatic models [8]. Our initial studies using ffLuc-eGFP LLC tumors transplanted into GH mice showed that in vivo BL increases within the range of 1.5×10^5^ to 5×10^7^ photon/sec/rad reliably represent metastatic growth following resection of subcutaneous tumors (Fig. S9A). Encouraged by the demonstrated ability of GH mice to detect therapeutic differences in metastatic disease, we tested a first-line chemotherapeutic drug in a post-resection adjuvant setting. Tumors from ffLuc-eGFP-labeled LLC were transplanted subcutaneously into syngeneic GH mice and resected at 500 mm^3^, after which mice were randomized to receive vehicle or gemcitabine. BL imaging showed that metastasis progressed efficiently in mice from the control treatment group (Fig. 6A), but was greatly suppressed by gemcitabine (Fig. 6B). Accordingly, gemcitabine significantly prolonged mouse disease-free survival (*P*<0.0001, time median undecided vs. 11 days in control; Fig. 6C). BL signals from *in vivo* imaging well corresponded to the metastatic nodules identified in harvested lungs by visual observation and *ex vivo* imaging (Fig. 6D). At the endpoint, the metastatic burden detected by in vivo BL imaging was also validated by ex vivo imaging of the harvested lungs (Fig. S10A). To determine if fluorescence could be exploited to isolate tumor cells for molecular analyses, whole lung single cell suspensions from untreated GH mice were subjected to FACS. The eGFP^+^ LLC cells were readily separated from all stromal cells by FACS (Fig. 6E), and formed well-labeled tumors upon re-transplantation (Fig. S10B).

## Discussion

Based on recent clinical breakthroughs in immunotherapy [30], and the ever-expanding evidence that the immune system plays numerous key roles in tumorigenesis, the need for immunocompetent preclinical mouse models has become acute. Immunocompetent GDA transplantation models offer significant advantages, allowing: incorporation of human-relevant genomic alterations and environmental insults into GEM-derived allografts; appropriate microenvironmental interactions between the transplanted tumor and host; preclinical and molecular analyses of metastatic lesions and perfectly matched sets of pre- and post-treatment samples; and industry-friendly experimental turnaround time. Immunocompromised patient-derived xenograft (PDX) models have shown promise as preclinical tools for testing chemotherapy [31], but the approach to modify host mice to bear a “humanized” immune system is prohibitively expensive and mostly untested.

The full value of any preclinical model can only be realized if cancerous lesions can be accurately monitored longitudinally. On balance optical reporters offer superior qualities and are widely used; unfortunately, their xenobiotic nature confounds their use in the context of a fully competent murine immune system. In fact, any xenobiotic gene introduced into immunocompetent animals poses a potential problem [32], [33], including other reporters [34], recombinases [35], transactivating factors [36] and viral oncogenes [37]. In this report we demonstrate that xenobiotic reporters induce problematic immune responses in immunocompetent mice, causing inconsistent activity and altered tumor behavior. We also describe a new GEM model immunologically tolerant to ffLuc and eGFP, which can serve as a transplantation host for any so-labeled syngeneic tumors. Immune responses induced by optical markers substantially affected growth, progression, and therapeutic responses of tumors transplanted into WT hosts, problems that were minimized or eliminated by using pre-tolerized GH mice. This difference was most notable with metastatic disease. GH mice enable consistent ffLuc-eGFP reporter activity, accurate monitoring throughout longitudinal studies, and tumor cell isolation for molecular analyses, all in the context of a normal immune system. Moreover, GEMs pre-tolerized to virtually any imageable marker can now be developed and exploited.

Most notably, immunity against reporter genes expressed in labeled tumors could significantly alter the outcome of preclinical therapeutic studies. Our first study showed that, relative to GH mice, paclitaxel delayed the growth of ffLuc-eGFP-expressing LLC tumors in WT hosts, where it induced a cytotoxic T cell response. Consistent with our observations, the immunogenicity of cell death induced by cytotoxic agents has been reported to be a critical determinant of chemotherapeutic efficacy [5]. However, we were surprised to observe that labeled tumors transplanted into WT mice could also be less responsive to drugs relative to those transplanted into GH hosts, indicating that the precise consequence of xenobiotic reporter expression is context-dependent (e.g. tumor type, tumor location, drug). The impact of such preclinical uncertainty on cancer patients is the possible inclusion of an ineffective drug *or* the exclusion of an efficacious drug in clinical trials. Therefore, results obtained from preclinical studies using labeled tumors transplanted into immunocompetent WT mice must be interpreted with great caution.

Interestingly, we found that reporter activity and growth of labeled transplanted tumors were altered not only in syngeneic WT, but also in partially immunocompromised NOD/SCID mice. Similarly, while progressing efficiently in GH mice, spontaneous metastasis was delayed or suppressed in NOD/SCID as well as WT mice. NOD/SCID mice are defective in adaptive immunity, but retain some innate immune function, including NK cell activity [38]. These findings suggest that xenobiotic reporters activate innate immunity, and indicate that immunocompromised mice with residual immunity cannot fully overcome the labeling inconsistency observed in WT mice.

The results above have demonstrated the complicated interaction between tumor antigens and immune system. The antibody reaction in WT vs. GH mice observed in Fig. 2D indicated that ffLuc-eGFP is an antigen capable of activating B cells. The results that tumor progression was delayed in NOD-SCID mice as compared to GH mice suggested that NK cells are involved, since the former still exhibits residual NK cell activity [38]. We further demonstrated that cytotoxic T cell response induced by ffLuc-eGFP induced was significantly enhanced by paclitaxel treatment (Fig. 6). Importantly, chemotherapy and targeted drug may modify the response against tumor antigen, as proposed by many studies [5]. The results above have suggested that immune system may respond to xenobiotic antigens in multiple, inter-dependent mechanisms, including adaptive (B and T cells) and innate (NK cells) immunity. In fact, a routine practice for the analysis of immune response is to compare antigenic responses between a specific mouse strain and a pre-tolerized control strain. In this regard, GH mice serve as “control” strain to study the immune response. Therefore, GH mice can also be a useful tool for immunological studies. Our complex immune system is involved to varying degrees in virtually all aspects of health and disease. Inclusion of an immune system in any preclinical model is clearly highly desirable, and of course essential when assessing highly promising immunotherapies. Preclinical cancer models become more valuable and versatile when tumor progression and drug response can be accurately and longitudinally monitored, an ability that represents an imposing challenge with the most relevant models where tumors are evaluated at orthotopic and/or metastatic sites. The Glowing Head mouse enables the consistent and reliable tracking of the progression and therapeutic response of tumors in the context of a normal immune system. We anticipate that the use of this GEM model will facilitate the assessment of metastatic and recurrent disease, permit the evaluation of immunomodulatory drugs both alone and in combination with small molecule inhibitors, and enhance the ability of preclinical models to predict clinical efficacy.

## Acknowledgments

We thank Drs. Rhonda Kineman (University of Illinois-Chicago, USA) for providing the rGH-Cre vector and Richard Palmiter (University of Washington, Seattle, WA) for his permission. We also thank Drs. Dominic Esposito (Leidos Biomedical Research, Inc., Frederick, MD, USA) for lentiviral vector production and Miriam Anver (Leidos Biomedical Research, Inc., Frederick, MD, USA) for histopathology. We are also grateful to Drs. Jude Alsarraj and Kent Hunter (National Cancer Institute, Bethesda, MD, USA) for providing the ffLuc-eGFP-labeled Mvt-1 breast cancer cells for the metastatic model. The content of this publication does not necessarily reflect the views or policies of the Department of Health and Human Services, nor does mention of trade names, commercial products, or organizations imply endorsement by the U.S. Government.

## Supporting Information

Figure S1 Expression of the ffLuc-eGFP reporter cannot be consistently maintained in labeled melanoma cells transplanted into strain-matched WT immunocompetent mice. **A**, Melanoma cells derived from mutant NRas-expressing p19ARF-null transformed mouse melanocytes were transplanted subcutaneously into isogenic F1 mice from C57BL/6 X 129 crosses, followed by periodic BL imaging and tumor measurement. The primary tumors were resected at day 25, and metastases were found in #38 and 39 the day after by imaging. The lungs were harvested from #38, and a single glowing metastatic nodule selected via the guidance of ex vivo imaging was transplanted into two isogenic mice in the second passage (P2). Imaging results showed that the reporter activity could not be consistently maintained in P2 mice, indicating that the inconsistency of reporter activity in immunocompetent mice could not be rescued by selection of a high-expressing tumor clone. **B**, Melanoma cells harvested from a HGF-transgenic/CDKN2A-knockout mouse were dissociated and transduced with the ffLuc-eGFP gene ex vivo, followed by subcutaneous transplantation of 10,000 cells into syngeneic FVB/N mice. The mice were periodically subjected to tumor measurement and bioluminescence (BL) imaging for reporter activity (upper panel). All tumors grew from day 14 to day 18 (lower left panel). However, BL intensity was reduced in #62 (red line) and extinguished in #63 (green line), while slowly increasing in the other three tumors (lower middle panel). The labeling retention of the tumors, measured as BL intensity/size ratio, was actually reduced in three of five tumors (lower right panel). These results indicate that ffLuc-eGFP activity in the labeled tumor could not be consistently maintained in syngeneic immunocompetent mice.
(TIF)

Figure S2 Generation of rGH-ffLuc-eGFP transgenic (GH) mouse. **A**, Selection of germline GH mice. Founders G6 and D8 were bred with wildtype (WT) mice to generate germline GH mice. In the examples of bioluminescence (BL) imaging shown here, pups #1–5 and #6–10 were generated from founder G6 and D8, respectively. Mouse #3 had head BL 2.3×106 photon/sec/rad, which is more than 20-fold higher than the WT background (1.0×105) shown in B. In contrast, #7 and #8 exhibited head BL 2–6 fold over WT background. Therefore, pups derived from Line D8 were selected for further breeding. This line shows stable transgene expression through generations. **B**, rGH targeted reporter gene expression to pituitary gland is highly specific. Sixteen GH and seven WT FVB/N mice from the same colonies used in this study were subjected to BL imaging for 1 min under anesthesia in ventral position. These results show the high specificity of BL signal in the head of GH mice.
(TIF)

Figure S3 Comparison of tumor labeling consistency in wildtype and GH mice. The ffLuc-eGFP-labeled LLC tumor selected from in vivo cycling in GH mice was transplanted into syngeneic wildtype (c-Brd) and GH mice. The tumor size (left panel; mm3 ± SE) and BL signal (right panel; photon/sec/rad ± SE) were measured periodically following transplantation. At day 10, no significant difference in tumor size was found between c-Brd and GH mice (p = 0.86). However, BL intensities from tumors in GH were significantly higher than that in c-Brd (p = 0.036).
(TIF)

Figure S4 BL images of mice from the study in Fig. 3A to 3D. **A**, Comparison of progression of ffLuc-eGFP-labeled mammary tumors in GH and WT mice. Mvt1 mouse breast cancer cells were transduced with ffLuc-eGFP-encoded lentivirus and the GFP+ tumor cells FACS isolated and expanded in culture. These cells were injected into mammary fat pads in syngeneic WT and GH FVB/N mice. Time as days after inoculation were indicated here. Images of primary tumors at day 7 and 11 and post-resection images from day 26 to 45 are shown here. D-number indicates the day that mouse morbidity was first diagnosed or noted (e.g. D26 is day 26). **B**, Metastatic pattern in WT and GH mice. At the endpoint, mice were injected with the luciferase substrate luciferin and euthanized. The internal organs were exposed and subjected to ex vivo imaging. In WT mice, metastases were detected almost exclusively in lungs (left panels). In GH mice, metastases were often detected in the thyroid, pleural, spleen, and/or peritoneum, as well as the lung (right panels).
(TIF)

Figure S5 BL images of mice from the study in Fig. 3E to 3G. Time as days after primary tumor resection are indicated. Post-resection images from day 3 to 14 are shown here. D-number indicates the day that mouse morbidity was first diagnosed or noted (e.g. D9 is day 9).
(TIF)

Figure S6 Responses of subcutaneous LLC tumors to paclitaxel within the dose range of 6.7–22.5 mg/kg. LLC cells from in vitro culture were inoculated subcutaneously. Upon reaching 125 mm^3^ at day 5, treatments with the indicated doses were initiated. A single dose was given each day for five days. Tumor sizes were measured by caliper. No significant efficacy was observed.
(TIF)

Figure S7 Representative hematoxylin and eosin staining of spleen sections from each treatment group. Note that spleens from the paclitaxel-treated WT c-Brd mice exhibited more lymphoid follicles (deep purple region) with disrupted structures, corresponding hematopoiesis and splenomegaly.
(TIF)

Figure S8 Pathological analyses of inflammation and invasion in melanoma allografts transplanted into GH and WT mice. **A–C**, representative images of tumors in GH mice receiving vehicle control, 50 mg/kg, or 100 mg/kg crizotonib (Criz). A, In vehicle control group, tumor invades into the deep subcutaneous tissue (arrows), but does not reach the level of the deep cutaneous skeletal muscle. Note that there are scattered mild inflammatory infiltrates throughout the deep subcutaneous tissue. B, Treatment of crizotinib at 50 mg/kg slightly reduced invasion into the deep subcutaneous adipose tissue (arrows) as compared to A. Mild to moderate inflammation surrounds this invasive front. C, In the treated group of 100 mg/kg crizotinib there are no distinct invasive foci, and mild inflammatory infiltrates are present along the tumor/subcutaneous tissue interface. **D–F**, representative images of tumors in WT mice receiving vehicle control, 50 mg/kg, or 100 mg/kg crizotonib. D, In vehicle control group, deep invasion into the underlying cutaneous skeletal muscle (arrows) can be observed in WT mice. However, this degree of invasion is very rare in GH mice. E, In the treated group of 50 mg/kg crizotinib deep invasive tumor foci are still observed (arrows), as well as large regions of dense inflamed granulation tissue (*), which was commonly observed at the deep invasive front in tumors in WT mice. F, In tumors from mice receiving 100 mg/kg crizotinib, dense granulation tissue (*) to the primary subcutaneous tumor, as well as a deeper invading melanoma (M) that contains abundant hemorrhage, are occasionally observed.
(TIF)

Figure S9 Correlation between disease burden and in vivo BL in the metastatic model. ffLuc-eGFP-labeled LLC tumors were subcutaneously transplanted into GH mice. Upon reaching 500 mm^3^, primary tumors were resected from mice, which were subjected to BL imaging periodically. Mice were selected at different in vivo BL intensities to be euthanized. The harvested lungs were fixed and sectioned for pathological analysis. **A**, H&E staining of lung sections from mice transplanted with fLuc-eGFP-labeled LLC tumors (the same used in Fig. 2-1). Under each panel is the chest BL intensity of each mouse from in vivo imaging. **B**, The disease burden in A was quantified with an Aperio slide image analysis system (Leica Biosystems). The correlation between in vivo BL signal and area of metastases in lung section follows a logarithmic function in regression analysis, a result similar to our previous study (Int. J. Cancer 2012, 130: 190–9).
(TIF)

Figure S10 Validation of the function of the metastatic model based on transplantation of labeled tumors into GH mice. **A**, At the endpoint of the study in Fig. 6, following in vivo BL imaging, the mice were euthanized, and the freshly harvested lungs were subjected to ex vivo BL and bright field imaging to identify metastatic nodules. The higher in vivo BL intensity was associated with either more or bigger sized nodules. The results validated quantitation by in vivo BL imaging. **B**, The GFP^+^ cells isolated in Fig. 6D and E were subcutaneously inoculated into three GH mice. After 7 days, the mice were subjected to tumor size measurement and BL imaging. The results showed that FACS-isolated cells were able to grow tumors.
(TIF)
