# Supplementary figures and images for: “Glowing Head” Mice: A Genetic Tool Enabling Reliable Preclinical Image-Based Evaluation of Cancers in Immunocompetent Allografts
Source: PLoS One. 2014 Nov 4;9(11):e109956. doi: 10.1371/journal.pone.0109956 (PMC4219677; doi:10.1371/journal.pone.0109956)

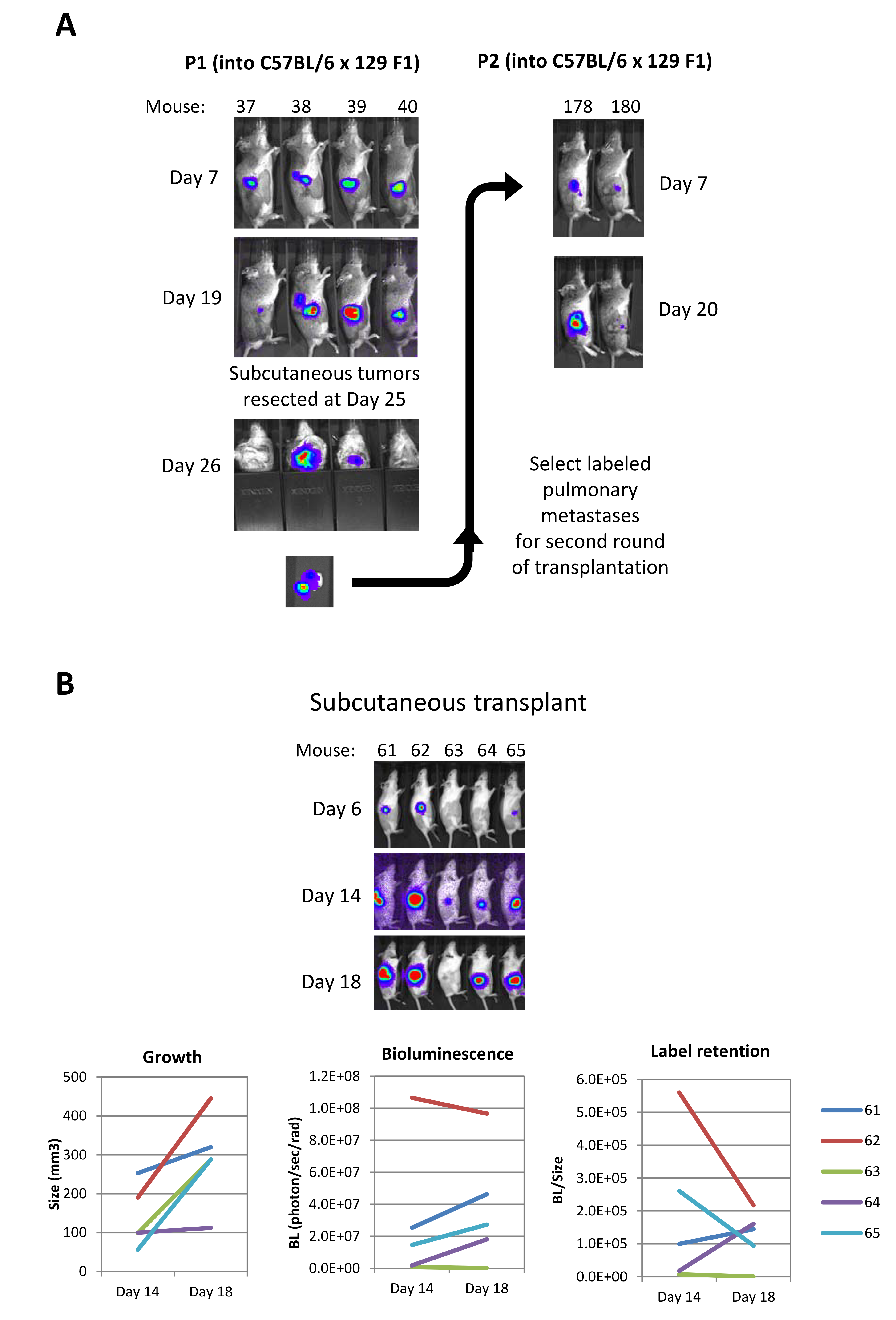

Supplement: Figure S1 — Expression of the ffLuc-eGFP reporter cannot be consistently maintained in labeled melanoma cells transplanted into strain-matched WT immunocompetent mice. A, Melanoma cells derived from mutant NRas-expressing p19ARF-null transformed mouse melanocytes were transplanted subcutaneously into isogenic F1 mice from C57BL/6 X 129 crosses, followed by periodic BL imaging and tumor measurement. The primary tumors were resected at day 25, and metastases were found in #38 and 39 the day after by imaging. The lungs were harvested from #38, and a single glowing metastatic nodule selected via the guidance of ex vivo imaging was transplanted into two isogenic mice in the second passage (P2). Imaging results showed that the reporter activity could not be consistently maintained in P2 mice, indicating that the inconsistency of reporter activity in immunocompetent mice could not be rescued by selection of a high-expressing tumor clone. B, Melanoma cells harvested from a HGF-transgenic/CDKN2A-knockout mouse were dissociated and transduced with the ffLuc-eGFP gene ex vivo, followed by subcutaneous transplantation of 10,000 cells into syngeneic FVB/N mice. The mice were periodically subjected to tumor measurement and bioluminescence (BL) imaging for reporter activity (upper panel). All tumors grew from day 14 to day 18 (lower left panel). However, BL intensity was reduced in #62 (red line) and extinguished in #63 (green line), while slowly increasing in the other three tumors (lower middle panel). The labeling retention of the tumors, measured as BL intensity/size ratio, was actually reduced in three of five tumors (lower right panel). These results indicate that ffLuc-eGFP activity in the labeled tumor could not be consistently maintained in syngeneic immunocompetent mice. (TIF) [file pone.0109956.s001.tif]

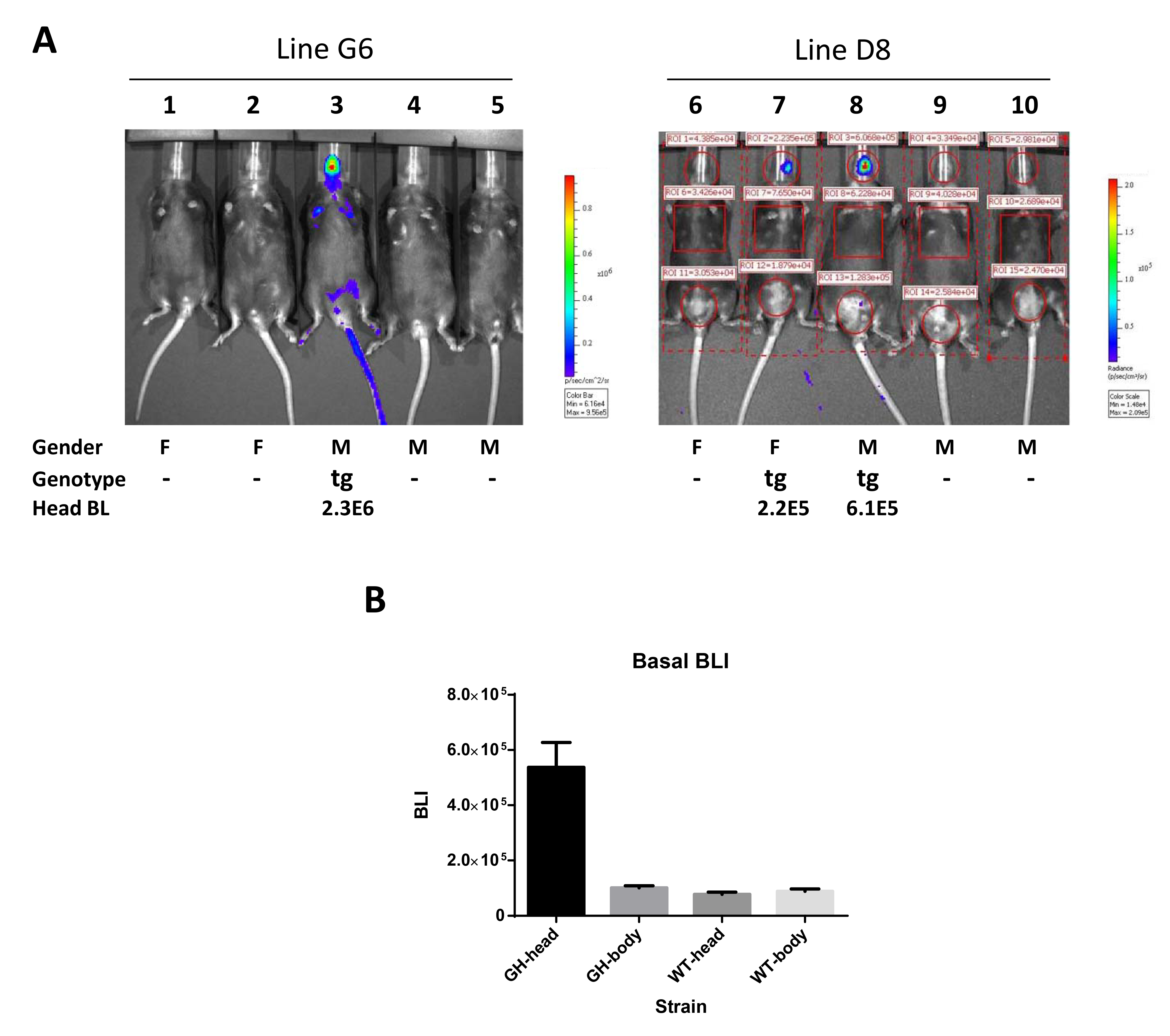

Supplement: Figure S2 — Generation of rGH-ffLuc-eGFP transgenic (GH) mouse. A, Selection of germline GH mice. Founders G6 and D8 were bred with wildtype (WT) mice to generate germline GH mice. In the examples of bioluminescence (BL) imaging shown here, pups #1–5 and #6–10 were generated from founder G6 and D8, respectively. Mouse #3 had head BL 2.3×10^6^ photon/sec/rad, which is more than 20-fold higher than the WT background (1.0×10^5^) shown in B. In contrast, #7 and #8 exhibited head BL 2–6 fold over WT background. Therefore, pups derived from Line D8 were selected for further breeding. This line shows stable transgene expression through generations. B, rGH targeted reporter gene expression to pituitary gland is highly specific. Sixteen GH and seven WT FVB/N mice from the same colonies used in this study were subjected to BL imaging for 1 min under anesthesia in ventral position. These results show the high specificity of BL signal in the head of GH mice. (TIF) [file pone.0109956.s002.tif]

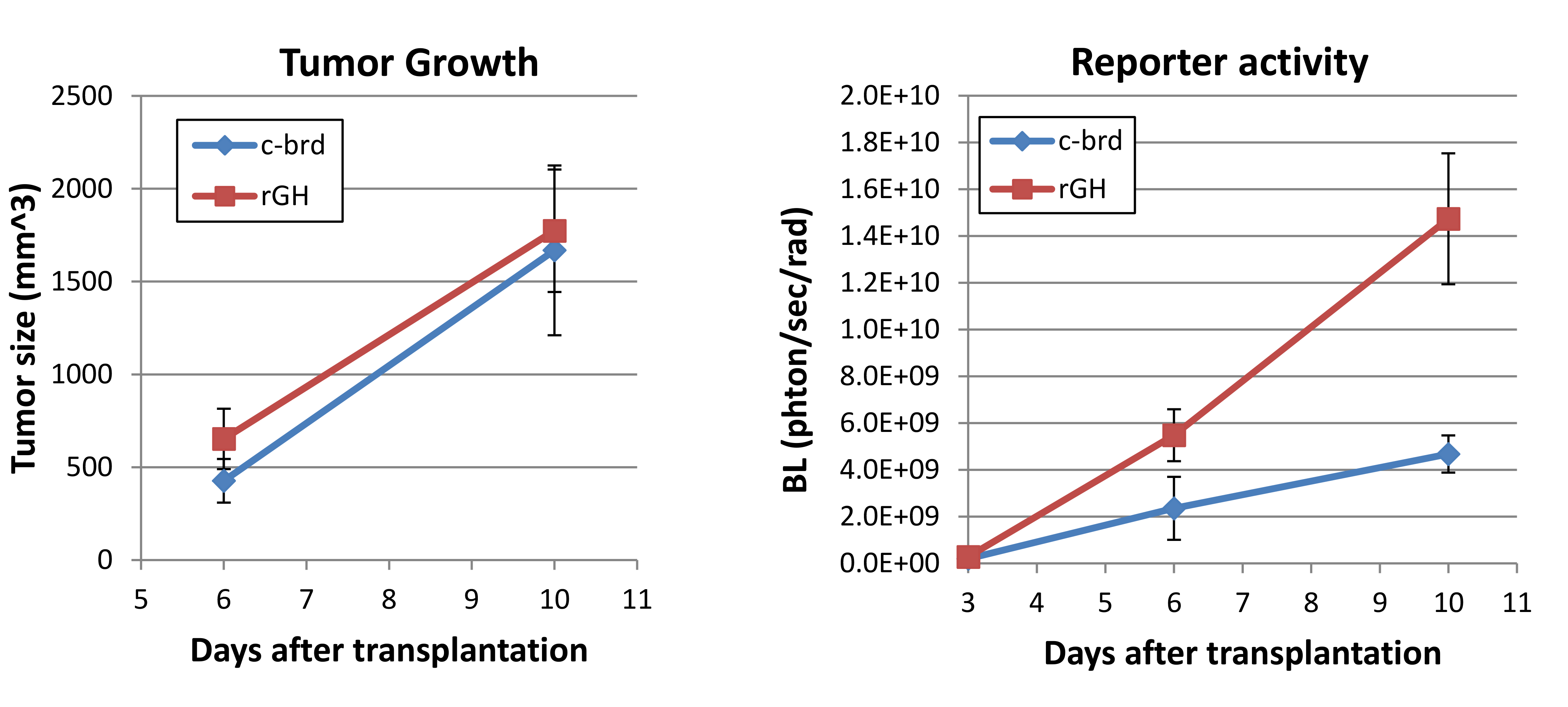

Supplement: Figure S3 — Comparison of tumor labeling consistency in wildtype and GH mice. The ffLuc-eGFP-labeled LLC tumor selected from in vivo cycling in GH mice was transplanted into syngeneic wildtype (c-Brd) and GH mice. The tumor size (left panel; mm3 ± SE) and BL signal (right panel; photon/sec/rad ± SE) were measured periodically following transplantation. At day 10, no significant difference in tumor size was found between c-Brd and GH mice (p = 0.86). However, BL intensities from tumors in GH were significantly higher than that in c-Brd (p = 0.036). (TIF) [file pone.0109956.s003.tif]

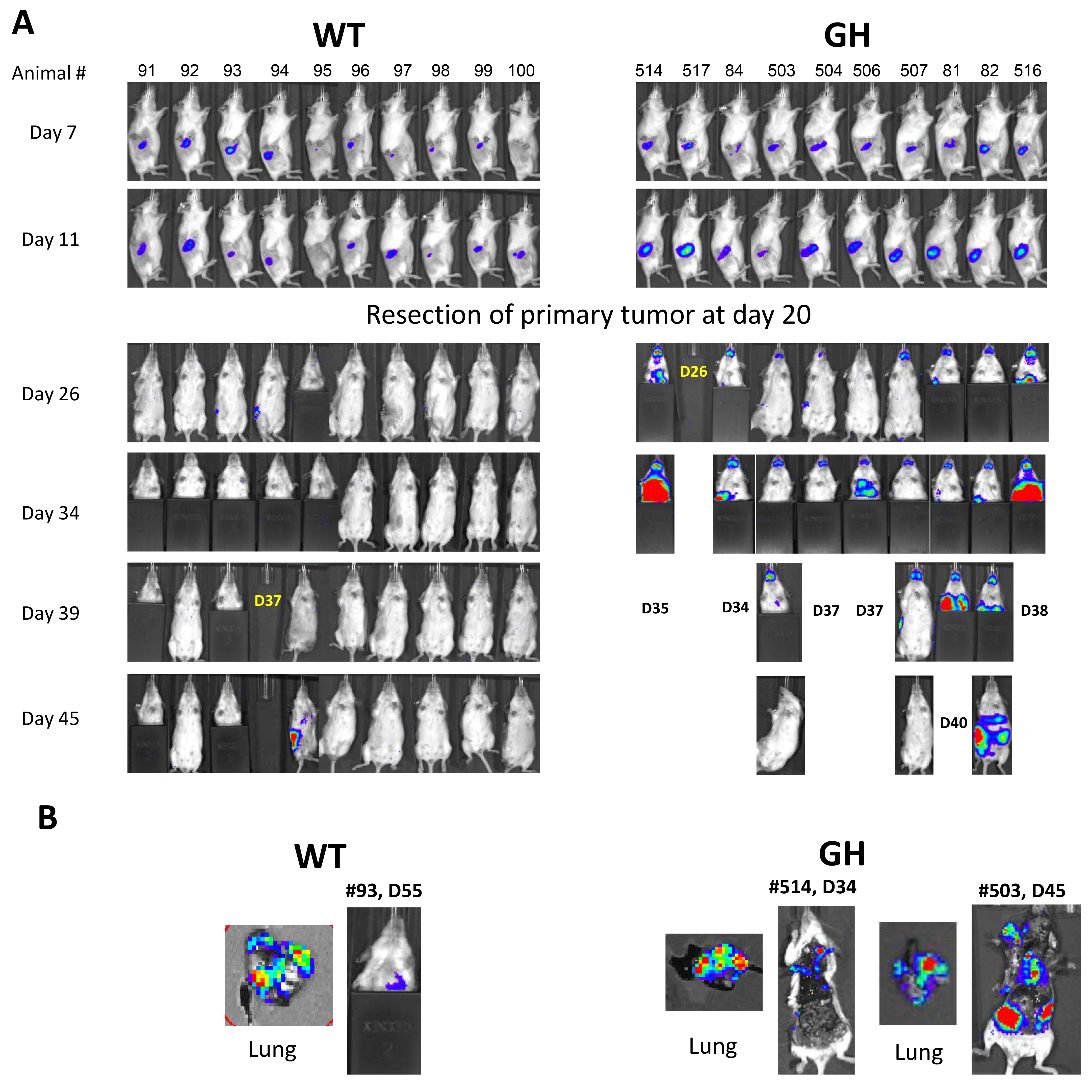

Supplement: Figure S4 — BL images of mice from the study in Fig. 3A to 3D. A, Comparison of progression of ffLuc-eGFP-labeled mammary tumors in GH and WT mice. Mvt1 mouse breast cancer cells were transduced with ffLuc-eGFP-encoded lentivirus and the GFP+ tumor cells FACS isolated and expanded in culture. These cells were injected into mammary fat pads in syngeneic WT and GH FVB/N mice. Time as days after inoculation were indicated here. Images of primary tumors at day 7 and 11 and post-resection images from day 26 to 45 are shown here. D-number indicates the day that mouse morbidity was first diagnosed or noted (e.g. D26 is day 26). B, Metastatic pattern in WT and GH mice. At the endpoint, mice were injected with the luciferase substrate luciferin and euthanized. The internal organs were exposed and subjected to ex vivo imaging. In WT mice, metastases were detected almost exclusively in lungs (left panels). In GH mice, metastases were often detected in the thyroid, pleural, spleen, and/or peritoneum, as well as the lung (right panels). (TIF) [file pone.0109956.s004.tif]

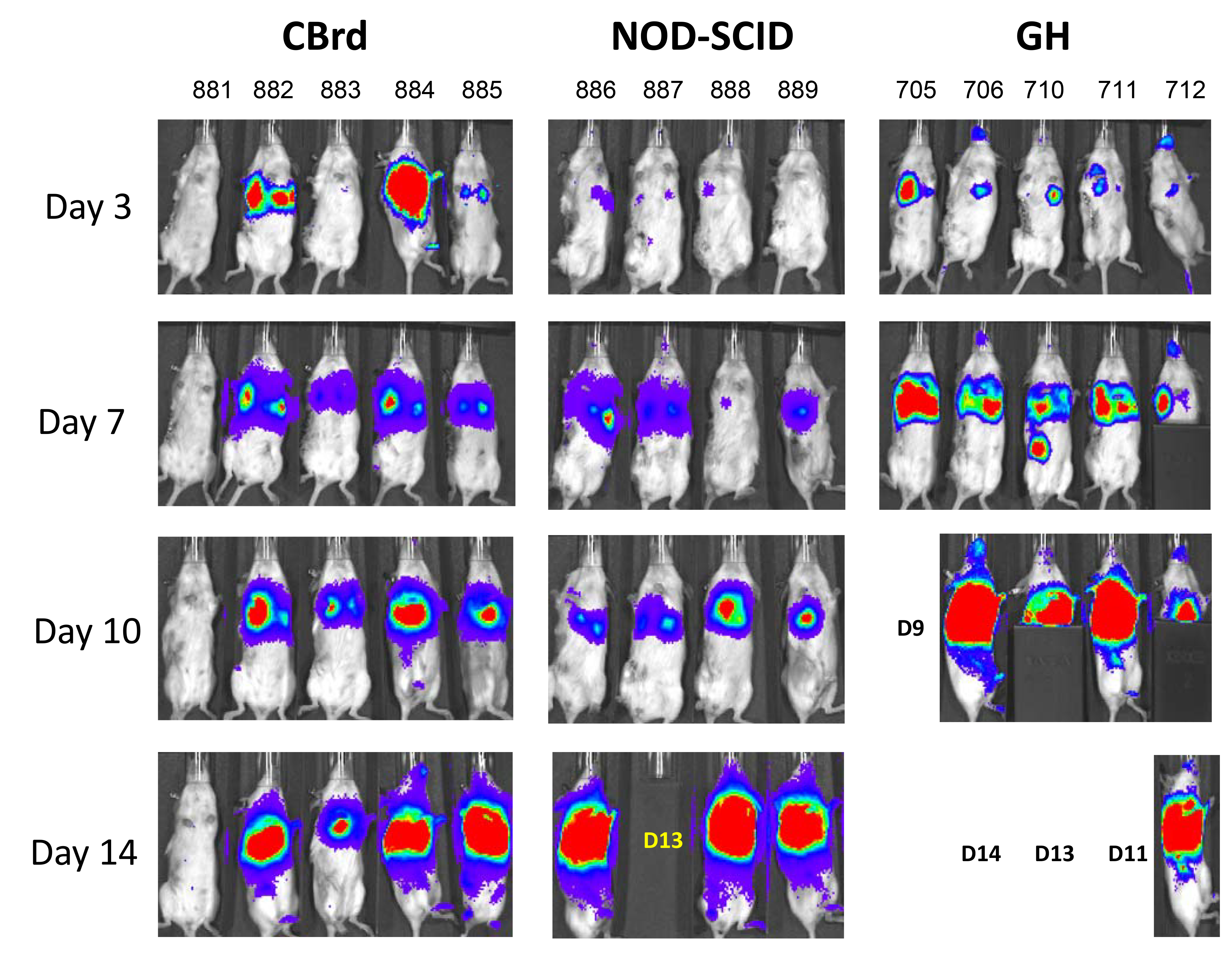

Supplement: Figure S5 — BL images of mice from the study in Fig. 3E to 3G. Time as days after primary tumor resection are indicated. Post-resection images from day 3 to 14 are shown here. D-number indicates the day that mouse morbidity was first diagnosed or noted (e.g. D9 is day 9). (TIF) [file pone.0109956.s005.tif]

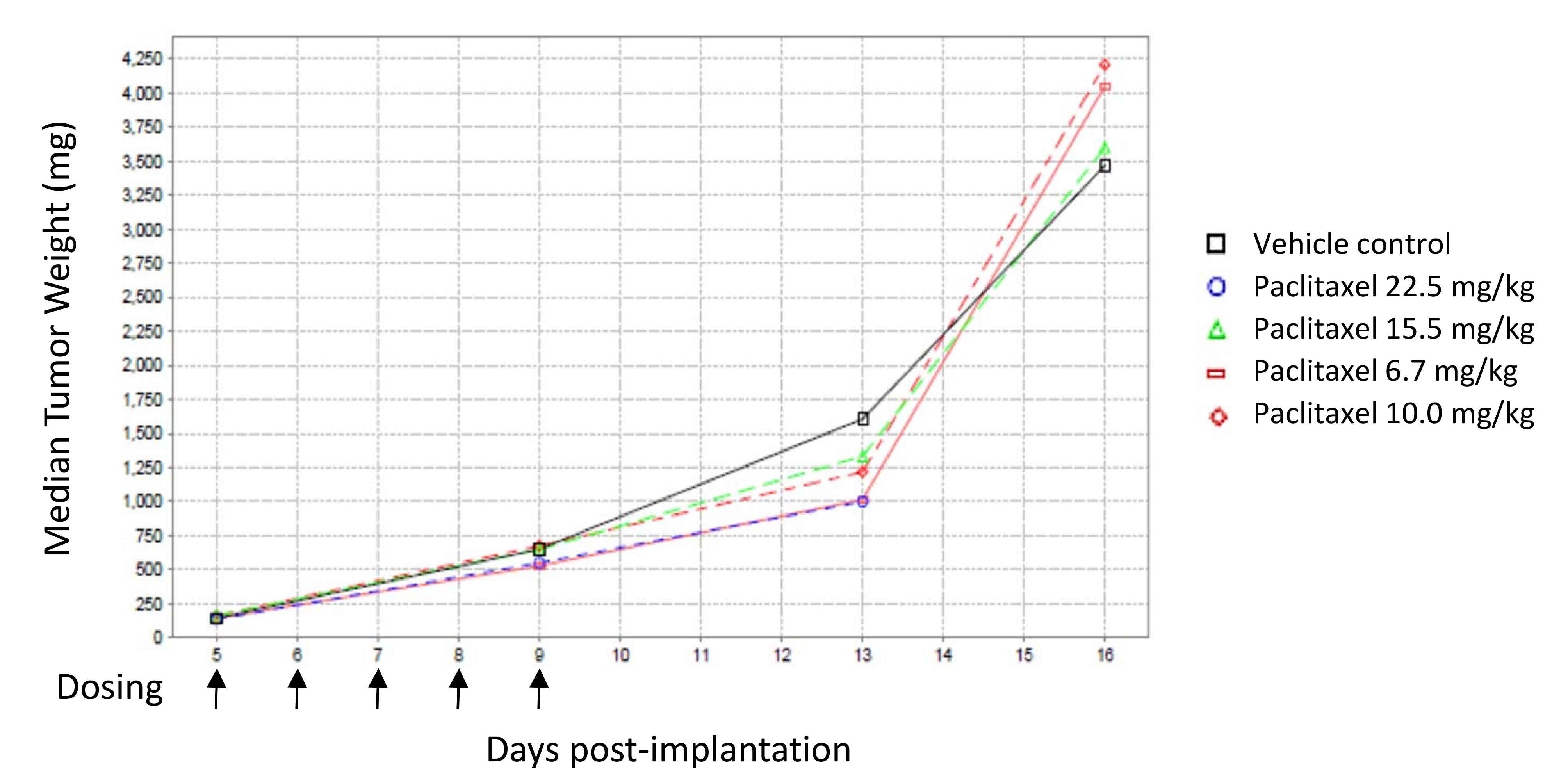

Supplement: Figure S6 — Responses of subcutaneous LLC tumors to paclitaxel within the dose range of 6.7–22.5 mg/kg. LLC cells from in vitro culture were inoculated subcutaneously. Upon reaching 125 mm3 at day 5, treatments with the indicated doses were initiated. A single dose was given each day for five days. Tumor sizes were measured by caliper. No significant efficacy was observed. (TIF) [file pone.0109956.s006.tif]

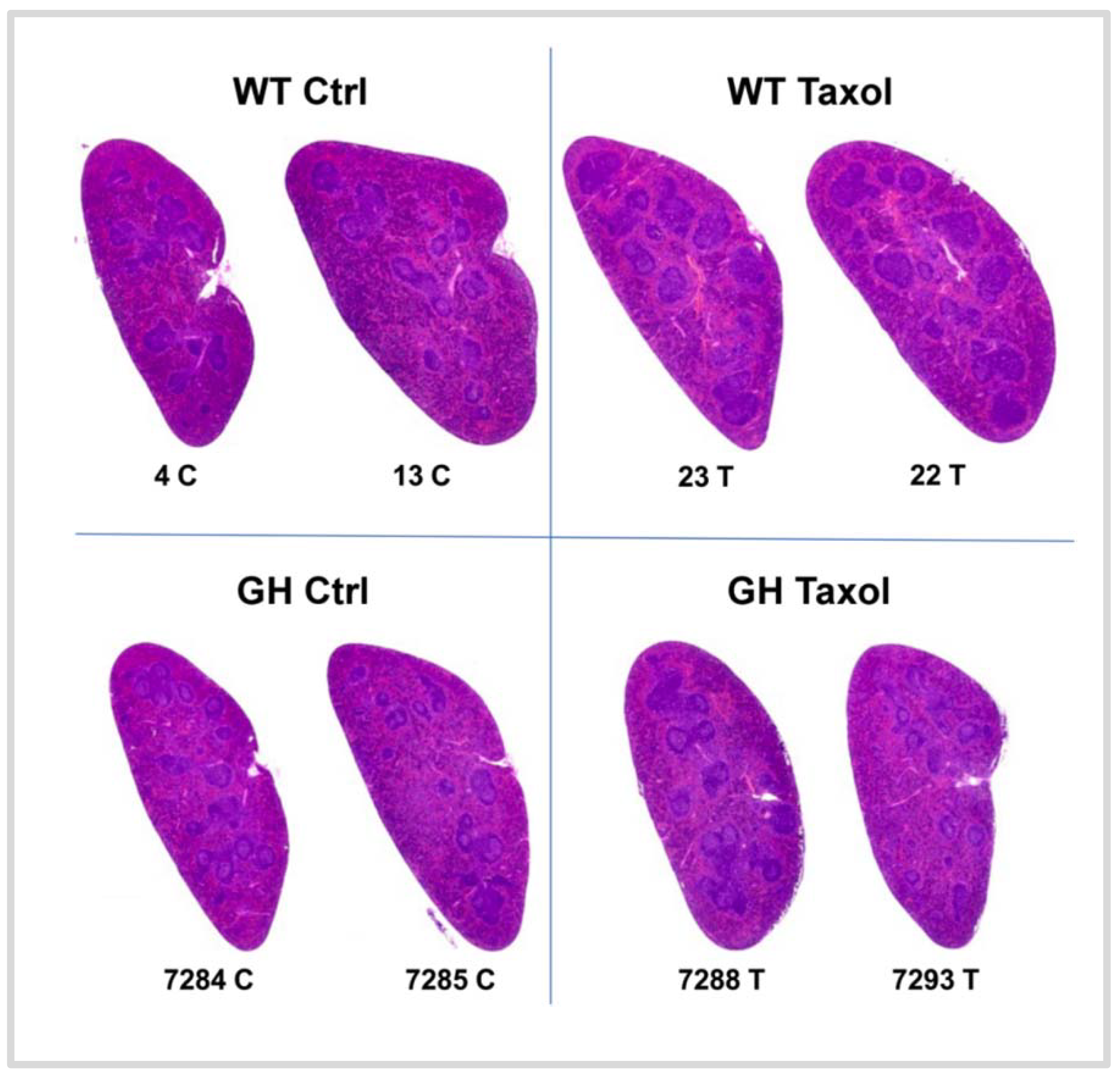

Supplement: Figure S7 — Representative hematoxylin and eosin staining of spleen sections from each treatment group. Note that spleens from the paclitaxel-treated WT c-Brd mice exhibited more lymphoid follicles (deep purple region) with disrupted structures, corresponding hematopoiesis and splenomegaly. (TIF) [file pone.0109956.s007.tif]

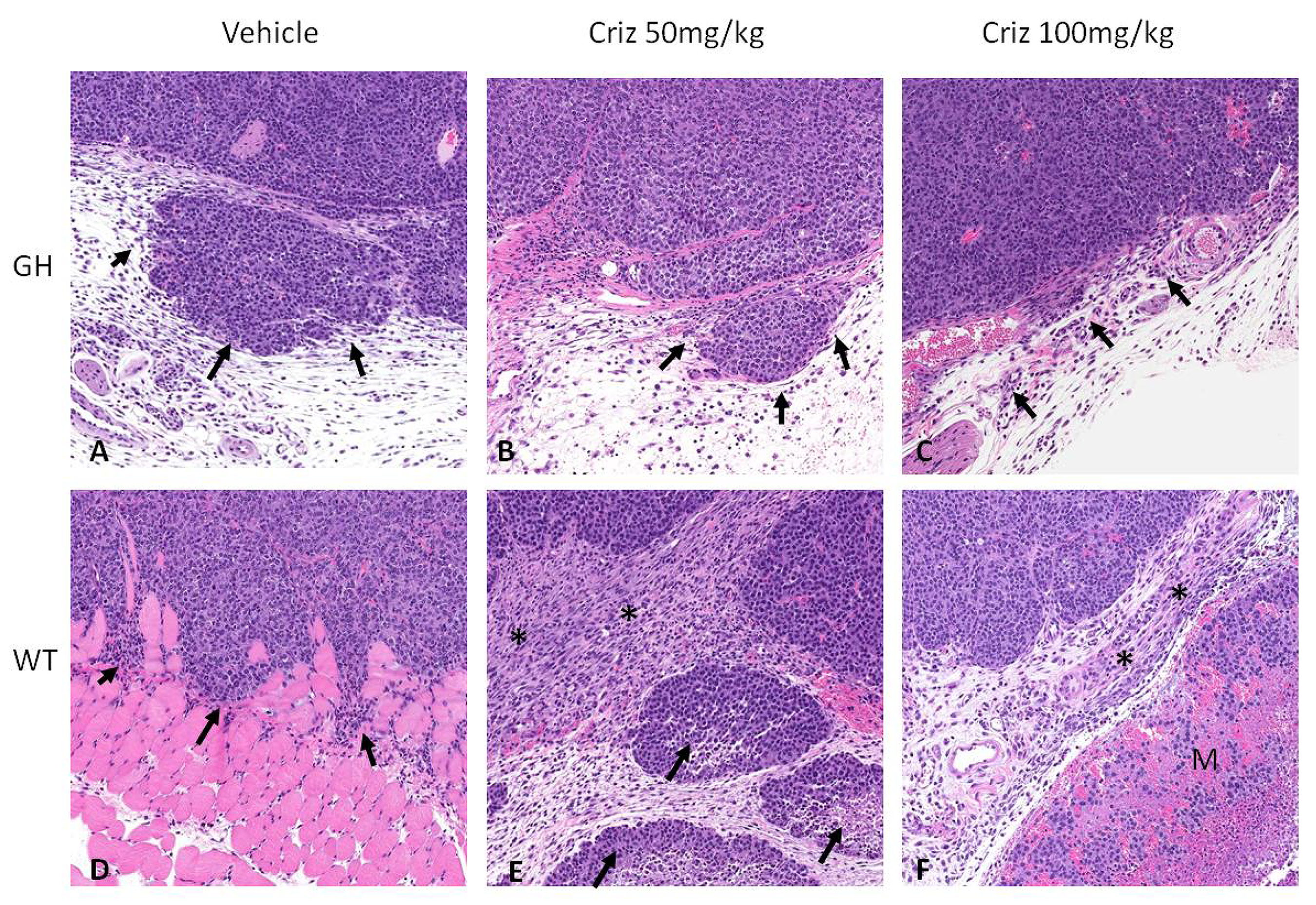

Supplement: Figure S8 — Pathological analyses of inflammation and invasion in melanoma allografts transplanted into GH and WT mice. A–C, representative images of tumors in GH mice receiving vehicle control, 50 mg/kg, or 100 mg/kg crizotonib (Criz). A, In vehicle control group, tumor invades into the deep subcutaneous tissue (arrows), but does not reach the level of the deep cutaneous skeletal muscle. Note that there are scattered mild inflammatory infiltrates throughout the deep subcutaneous tissue. B, Treatment of crizotinib at 50 mg/kg slightly reduced invasion into the deep subcutaneous adipose tissue (arrows) as compared to A. Mild to moderate inflammation surrounds this invasive front. C, In the treated group of 100 mg/kg crizotinib there are no distinct invasive foci, and mild inflammatory infiltrates are present along the tumor/subcutaneous tissue interface. D–F, representative images of tumors in WT mice receiving vehicle control, 50 mg/kg, or 100 mg/kg crizotonib. D, In vehicle control group, deep invasion into the underlying cutaneous skeletal muscle (arrows) can be observed in WT mice. However, this degree of invasion is very rare in GH mice. E, In the treated group of 50 mg/kg crizotinib deep invasive tumor foci are still observed (arrows), as well as large regions of dense inflamed granulation tissue (*), which was commonly observed at the deep invasive front in tumors in WT mice. F, In tumors from mice receiving 100 mg/kg crizotinib, dense granulation tissue (*) to the primary subcutaneous tumor, as well as a deeper invading melanoma (M) that contains abundant hemorrhage, are occasionally observed. (TIF) [file pone.0109956.s008.tif]

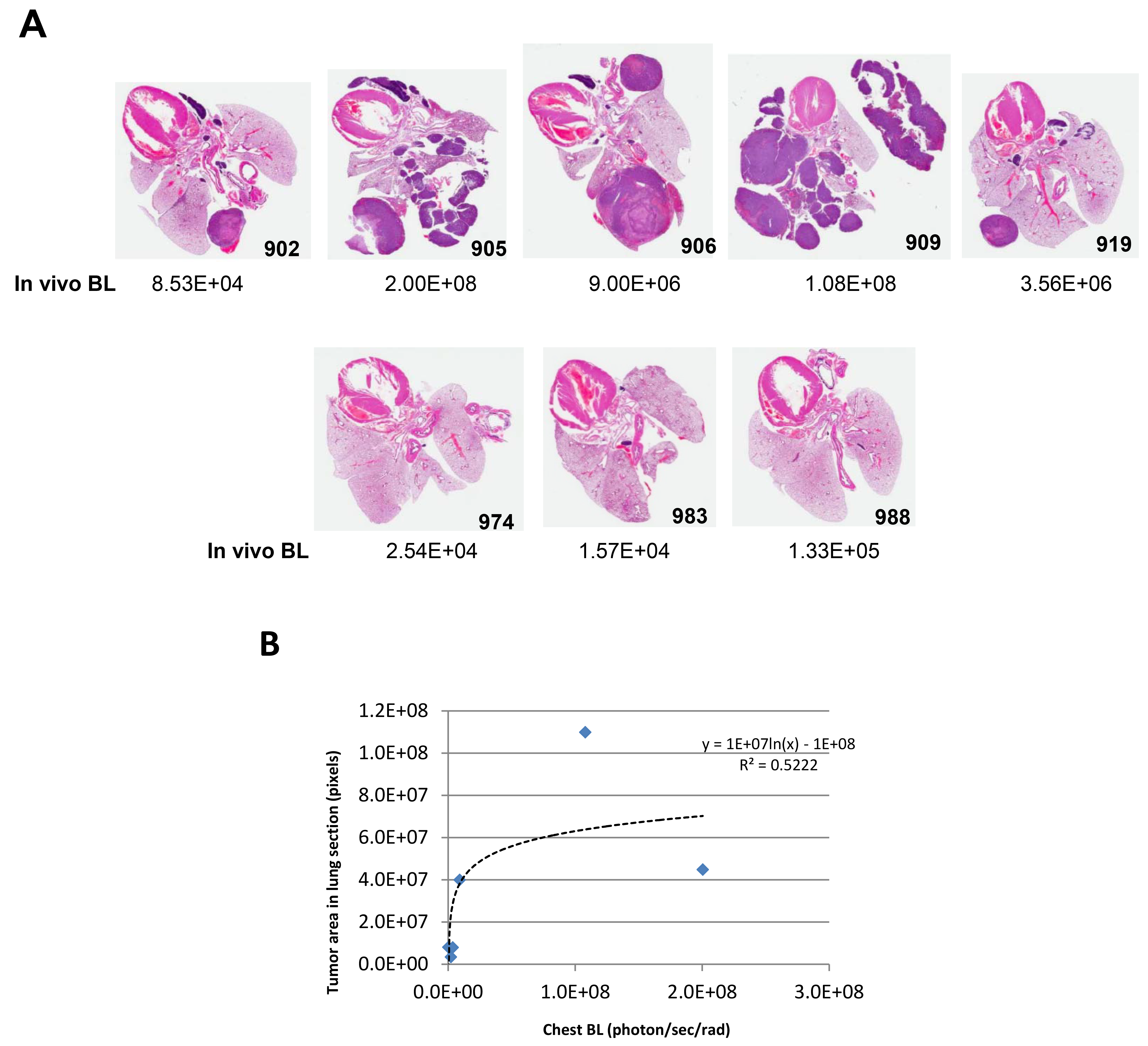

Supplement: Figure S9 — Correlation between disease burden and in vivo BL in the metastatic model. ffLuc-eGFP-labeled LLC tumors were subcutaneously transplanted into GH mice. Upon reaching 500 mm3, primary tumors were resected from mice, which were subjected to BL imaging periodically. Mice were selected at different in vivo BL intensities to be euthanized. The harvested lungs were fixed and sectioned for pathological analysis. A, H&E staining of lung sections from mice transplanted with fLuc-eGFP-labeled LLC tumors (the same used in Fig. 2-1). Under each panel is the chest BL intensity of each mouse from in vivo imaging. B, The disease burden in A was quantified with an Aperio slide image analysis system (Leica Biosystems). The correlation between in vivo BL signal and area of metastases in lung section follows a logarithmic function in regression analysis, a result similar to our previous study (Int. J. Cancer 2012, 130: 190–9). (TIF) [file pone.0109956.s009.tif]

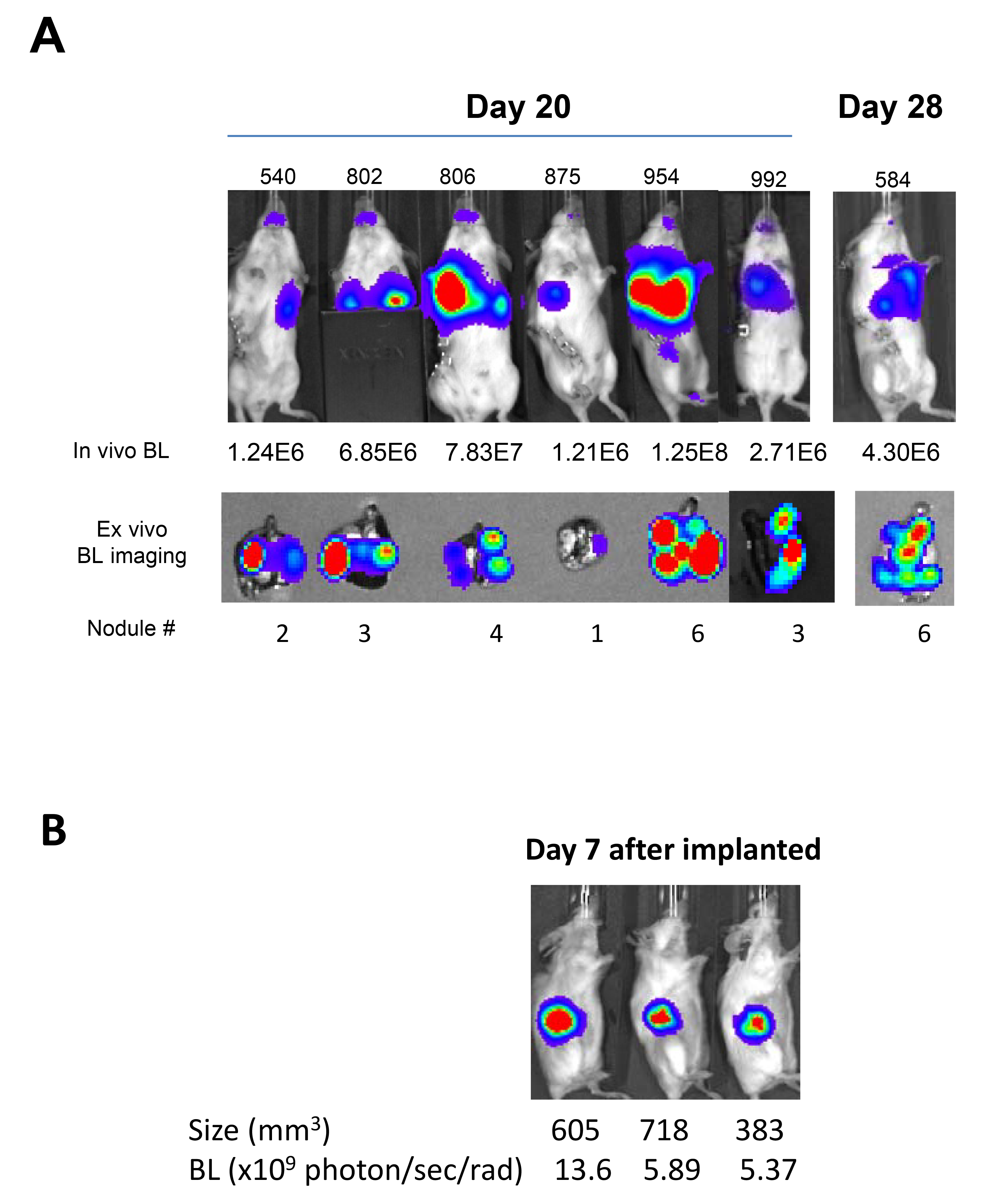

Supplement: Figure S10 — Validation of the function of the metastatic model based on transplantation of labeled tumors into GH mice. A, At the endpoint of the study in Fig. 6, following in vivo BL imaging, the mice were euthanized, and the freshly harvested lungs were subjected to ex vivo BL and bright field imaging to identify metastatic nodules. The higher in vivo BL intensity was associated with either more or bigger sized nodules. The results validated quantitation by in vivo BL imaging. B, The GFP+ cells isolated in Fig. 6D and E were subcutaneously inoculated into three GH mice. After 7 days, the mice were subjected to tumor size measurement and BL imaging. The results showed that FACS-isolated cells were able to grow tumors. (TIF) [file pone.0109956.s010.tif]
